# Pore formation in regulated cell death

**DOI:** 10.15252/embj.2020105753

**Published:** 2020-10-30

**Authors:** Hector Flores‐Romero, Uris Ros, Ana J Garcia‐Saez

**Affiliations:** ^1^ Institute for Genetics and Cologne Excellence Cluster on Cellular Stress Responses in Aging‐Associated Diseases (CECAD) University of Cologne Cologne Germany

**Keywords:** apoptosis, cell death, membrane pores, necroptosis, pyroptosis, Autophagy & Cell Death, Immunology

## Abstract

The discovery of alternative signaling pathways that regulate cell death has revealed multiple strategies for promoting cell death with diverse consequences at the tissue and organism level. Despite the divergence in the molecular components involved, membrane permeabilization is a common theme in the execution of regulated cell death. In apoptosis, the permeabilization of the outer mitochondrial membrane by BAX and BAK releases apoptotic factors that initiate the caspase cascade and is considered the point of no return in cell death commitment. Pyroptosis and necroptosis also require the perforation of the plasma membrane at the execution step, which involves Gasdermins in pyroptosis, and MLKL in the case of necroptosis. Although BAX/BAK, Gasdermins and MLKL share certain molecular features like oligomerization, they form pores in different cellular membranes via distinct mechanisms. Here, we compare and contrast how BAX/BAK, Gasdermins, and MLKL alter membrane permeability from a structural and biophysical perspective and discuss the general principles of membrane permeabilization in the execution of regulated cell death.

## Graphical abstract

In this review, Ana Garcia‐Saez and colleagues compare and contrast the mechanisms by which proteins such as BAX/BAK, Gasdermins and MLKL can create membrane pores, and which roles these play, in different forms of regulated cell death.

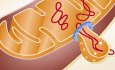

## Pore formation in membranes is a conserved strategy to kill cells

Biological membranes define biochemical environments and are fundamental for the existence of life (Deamer, 2016). Selective transport between these enclosed spaces is highly regulated and sustained disruption in membrane integrity is, as a consequence, a point of no return in cell death (Youle & Strasser, 2008; Kunzelmann, 2016; Broz *et al*, 2020). Yet, alterations in membrane permeability are relevant for a large number of biological processes including immunity, metabolism, and infection (Kagan, 2012; McCormack *et al*, 2013).

A membrane pore can be defined as any local membrane perturbation that allows the passive flow of molecules (Schwarz & Robert, 1992). Pore‐forming proteins (PFPs) represent a large and structurally diverse family of proteins that have the common function of altering membrane permeability by creating pores. They can act exogenously as secreted soluble proteins that permeabilize the plasma membrane of their target cells. This includes most pore‐forming toxins (PFTs), which are some of the most potent virulence factors found in nature (Ros & Garcia‐Saez, 2015; Dal Peraro & van der Goot, 2016) or perforin, which is released by cytotoxic T cells and Natural Killer cells (Voskoboinik *et al*, 2015; Prinz *et al*, 2020). PFPs can also be intracellular executioners as components of cell death signaling pathways (Espiritu *et al*, 2019; Flores‐Romero *et al*, 2020). For instance, Bcl‐2‐associated X protein (BAX) and BCL2‐antagonist/killer 1 (BAK) form pores that lead to mitochondrial outer membrane permeabilization (MOMP) during intrinsic apoptosis. Gasdermins (GSDMs) execute pyroptosis by a mechanism that culminates with pore opening at the plasma membrane. In necroptosis, mixed lineage kinase domain‐like (MLKL) induces plasma membrane permeabilization through a yet unclear mechanism that could also be linked to pore formation (Liu *et al*, 2016; Cosentino & Garcia‐Saez, 2017; Flores‐Romero & Garcia‐Saez, 2019a).

**Figure 1:**
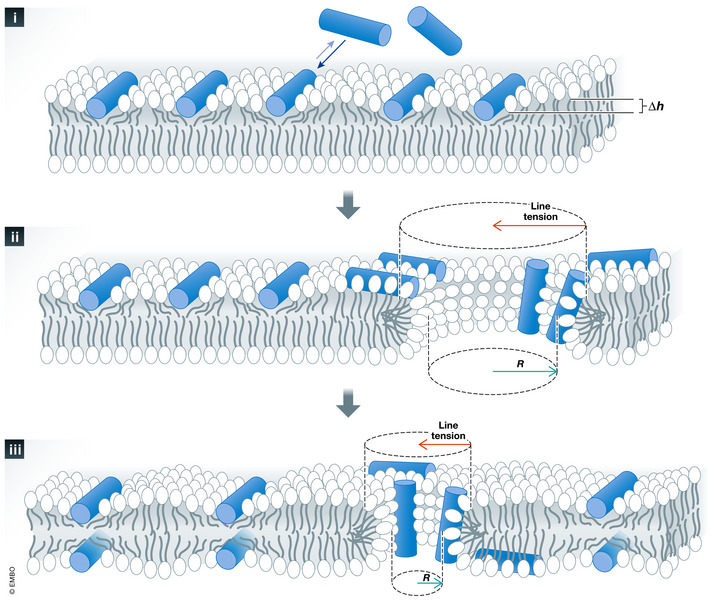
Mechanism of pore formation by cationic amphipathic peptides to exemplify the formation and stabilization of a toroidal pore (i) Pore‐forming peptides bind avidly to the accessible interface of the lipid bilayer and occupy a volume only in the interfacial region, which causes asymmetric stretching and membrane thinning (∆h). As a consequence, the membrane is stressed and destabilized, so that defects in the lipid bilayer become more likely and eventually a pore is formed (ii). Once the pore is open, a line tension appears at the pore edge due to the extra energy cost associated with the reorientation of the lipids into a highly curved boundary. This tension increases with the pore perimeter and is therefore a line tension. The initial pore grows quickly as long as the membrane tension dominates. But as the pore size grows, so does the counterbalancing line tension too. Furthermore, with the open pore, the peptides redistribute in the membrane by diffusion through the pore to the other monolayer, which reduces the membrane tension due to asymmetric distribution of the peptides. At a certain moment, the line tension becomes predominant and the pore size starts to decrease. However, if the pore‐forming peptides bound near the pore rim are able to reduce the line tension, an equilibrium can be reached with a smaller but stable pore (iii). R = radius, ∆h = change in the thickness of the membrane (absence vs. presence of the protein/peptide mass). Adapted from (Fuertes *et al*, 2011).

PFPs are usually classified in α or β, depending on the secondary structure of the protein segments forming the pores (Ros & Garcia‐Saez, 2015; Cosentino *et al*, 2016). In addition, PFPs can build different types of pores, as defined by the presence or absence of lipids in the pore structure (Ros & Garcia‐Saez, 2015; Gilbert, 2016). They are classified as protein‐lined if they are constituted only of proteins, as pure lipid pores, or as protein/lipid pores, when they contain both types of molecules. In protein‐lined pores, the lumen is solely covered by transmembrane segments of proteins organized into α‐ or β‐barrel “walls” (Dal Peraro & van der Goot, 2016; Gilbert, 2016). Importantly, in this type of pores, the membrane or certain membrane lipids can play a functional role in protein recruitment, assembly, and folding (Rojko & Anderluh, 2015; Gilbert, 2016). In contrast, membrane lipids together with the amphipathic regions of proteins or peptides form the edge of protein/lipid pores (Ludtke *et al*, 1996; Matsuzaki *et al*, 1996). Here, lipids rearrange from their bilayer distribution to a non‐lamellar assembly that creates a continuous surface in which the membrane bends at the pore boundary like a torus. Protein/lipid or toroidal pore opening is promoted by proteins or protein fragments that generate membrane tension. PFP accumulation at or next to the pore rim then reduces the line tension and stabilizes the open pore state (Karatekin *et al*, 2003; Puech *et al*, 2003) (Fig 1). Pure lipid pores are also toroidal, but in the absence of proteins, their opening probability and lifetime are very low. They mainly occur upon strong membrane perturbations such as mechanical and electrical tension or osmotic swelling (Tieleman *et al*, 2003; Tieleman & Marrink, 2006).

In regulated cell death, a diverse repertoire of endogenous pro‐death effectors reveals a plethora of strategies evolved to permeabilize cellular membranes. The architecture of the resulting pores is defined by specific protein and lipid compositions, as well as by intramolecular interactions. This determines pore heterogeneity, size, and stability, and thereby the type and extent of molecules that can be released through them. As a consequence, the properties of membrane pores impact the signaling cascades that are activated downstream of membrane permeabilization.

## BAX and BAK pores in apoptosis: old friends with new habits

### The toroidal pore of BAX and BAK

Currently, it is well established that the BCL2 family members BAX, BAK, and perhaps BOK are the executioners of MOMP and thereby fundamental effectors of the intrinsic apoptotic pathway (Moldoveanu & Czabotar, 2019). In healthy cells, BAX and BAK exist as inactive, monomeric proteins that constitutively shuttle between cytosol and mitochondria, with BAX being mostly cytosolic and BAK mitochondria‐associated (Edlich *et al*, 2011; Schellenberg *et al*, 2013; Todt *et al*, 2015; Lauterwasser *et al*, 2016). Upon apoptosis induction, both proteins accumulate at and insert into the mitochondrial membrane, undergo conformational rearrangements, oligomerize and form pores that release pro‐apoptotic factors such as cytochrome c and SMAC/DIABLO (Nechushtan *et al*, 2001; Rehm *et al*, 2003; Zhou *et al*, 2005; Edlich *et al*, 2011). However, the relative order of events, the structural intermediates involved, as well as the molecular properties of the membrane openings mediated by BAX‐type proteins remains controversial (Moldoveanu & Czabotar, 2019; Flores‐Romero & Garcia‐Saez, 2019a).

Despite their functional heterogeneity, the 3D structures of all multidomain BCL2 proteins (including not only BAX, BAK, and BOK, but also the pro‐survival family members) present the same globular α‐helical fold, with a predominantly hydrophobic central hairpin that is flanked on both sides by pairs of amphipathic α‐helices. This peculiar folding is strikingly similar to the pore‐forming domain of bacterial α‐PFTs such as the colicins and diphtheria toxin (Muchmore *et al*, 1996; Suzuki *et al*, 2000; Petros *et al*, 2004; Moldoveanu *et al*, 2013; Ke *et al*, 2018). Considering these structural similarities and their ability to allow currents through artificial membrane systems known as black lipid membranes, it was initially proposed that BAX‐type proteins induce MOMP by generating membrane pores or channels (Minn *et al*, 1997; Sattler *et al*, 1997; Schlesinger *et al*, 1997; Basanez *et al*, 1999).

**Figure 2:**
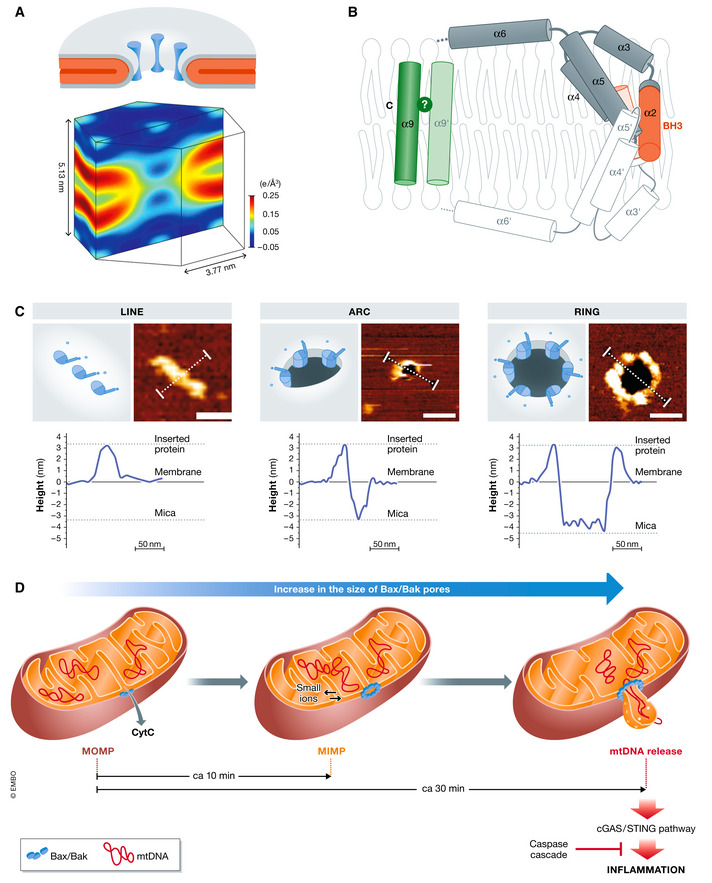
BAX/BAK toroidal pore (A) Schematic representations of the protein/lipid model shown as a 3D view cut through the membrane pore. Gray layers represent lipid headgroups of the bilayer, the acyl chains are shown in red and protein helices by dark cylinders (top). The corresponding normalized electron density distributions of acyl chains in lipid bilayers containing BAX α5 (bottom). Note that, unlike in a protein channel, in a toroidal pore: (i) the surface of the pore is lined by lipid headgroups, (ii) membrane monolayers are bent at the pore edge, and (iii) the two leaflets of the bilayer become continuous. Taken from (Qian *et al*, 2008). (B) Structural representation of membrane‐embedded BAX in the context of a toroidal pore based on (Bleicken *et al*, 2014; Bleicken *et al*, 2018). BAX is represented with nine cylinders corresponding to its nine helices. BH3 domain and C‐terminal/tail anchoring domain are depicted in orange and green, respectively. One monomer is shown in gray (1–9) and the other is depicted in white (1ʹ–9ʹ). The relative orientation of the helices α9 remains unresolved. (C) BAX oligomers are organized into line, arc, and rings. Each panel shows the schematic representation (left) and the AFM images (right) of BAX assemblies. Both arcs and rings but not lines, reveal a circular depression (black) that spans the lipid membrane (dark orange). BAX molecules around the pore rim (yellow) protrude above the membrane plane, as confirmed by the height cross‐sections shown below each image (corresponding to the white line in the AFM images). The topography of the arc structure reveals a pore only partially surrounded by BAX molecules, while lipids alone form the rest of the pore rim. Based on (Salvador‐Gallego *et al*, 2016). (D) Model for the temporal control of content release during MOMP. Upon apoptotic stimuli, BAX and BAK permeabilize the MOM and induce the release of apoptotic factors, for example, cyt C. The consequent MIM permeabilization and the widening of BAX/BAK pores induce the release of mtDNA in the cytosol. In absence of caspase activity, this leads to the activation of the cGAS/STING signaling pathway. Based on (Cosentino & Garcia‐Saez, 2018).

Different observations have since then supported the hypothesis that active BAX and BAK form long‐lived toroidal pores. *In vitro*, BAX decreases the lifetime of planar membranes and forms pores with variable conductance (Basanez *et al*, 1999) that are affected by the physical properties of the membrane and the presence of lipids with intrinsic monolayer curvature (Basanez *et al*, 2002; Terrones *et al*, 2004). A peptide derived from helix α5 of BAX forms membrane pores with lipid molecules in the lumen as demonstrated by X‐ray diffraction (Qian *et al*, 2008) and conductance experiments in black lipid membranes (Garcia‐Saez *et al*, 2005). This is in agreement with the transbilayer lipid movement coupled with membrane permeabilization induced by BAX (Epand *et al*, 2003; Garcia‐Saez *et al*, 2006) (Fig 2A and B). These lines of evidence converge into the current view of a protein/lipid pore of tunable size constituted by BAX/BAK homodimers, where the size of BAX and BAK pores is not constant, but it evolves with time and depends on protein concentration. In this model, the central hairpin of helices α5 and α6 lies on the membrane surface at the edge of the pore (Qian *et al*, 2008; Basanez *et al*, 2012; Bleicken *et al*, 2013; Mandal *et al*, 2016; Cosentino & Garcia‐Saez, 2017; Bleicken *et al*, 2018) (Fig 2A and B). Not only BAX/BAK molecules, but also the mechanical properties of the membrane play a role in the size and stability of the pores (Karatekin *et al*, 2003).

According to the toroidal pore model, the initial asymmetric insertion of BAX α‐helices into the cytosolic leaflet of the MOM stresses the membrane and generates membrane tension. Once a threshold tension is reached (which may be locally enhanced by protein concentration or oligomerization), the energy required to reorganize lipids out of the bilayer structure becomes thermally accessible and a pore opens, which dissipates membrane tension (Lee *et al*, 2004). Lipids reassemble into a torus around the membrane pore to avoid exposure of the hydrophobic acyl chains to the water environment. The two membrane monolayers form a continuous surface at the pore edge with negative curvature in the plane of the membrane and positive curvature in the plane perpendicular to the membrane. The bending of the lipids at the pore rim has an energetic cost that is directly proportional to the length of the pore, giving rise to line tension (Ludtke *et al*, 1996; Matsuzaki *et al*, 1996). Line tension acts as the driving force for pore closure and opposes membrane tension. As a result, toroidal pores are metastable structures whose lifetime is governed by the balance between membrane tension and line tension (Valcarcel *et al*, 2001; Yang *et al*, 2001; Karatekin *et al*, 2003; Fuertes *et al*, 2010b). In support of this model, the rim of BAX pores is formed by both lipid headgroups and protein molecules (Kuwana *et al*, 2016; Salvador‐Gallego *et al*, 2016), with BAX molecules decreasing the line tension to maintain the pore stably open (Basanez *et al*, 1999; Garcia‐Saez *et al*, 2007; Fuertes *et al*, 2010a; Fuertes, 2011; Unsay *et al*, 2017; Bleicken *et al*, 2018) (Figs 1 and 2B).

### BAX/BAK membrane topology in the context of the toroidal pore

Once in the membrane, activated‐BAX/BAK undergoes a rearrangement of their globular fold that implies first the opening of their N‐terminal region and then, in the case of BAX, the dislodgment and transmembrane insertion of the tail anchoring domain in helix α9 (Nechushtan *et al*, 1999; Griffiths *et al*, 2001). This is followed by exposure of their BH3 domain, which is thought to occur concomitantly with the reorganization of BAX/BAK into two functionally different regions, namely the dimerization/core (helices α2–α5) and the piercing/latch (helices α6–α8) domains (Dewson *et al*, 2008; Dewson *et al*, 2012; Czabotar *et al*, 2013; Moldoveanu *et al*, 2013; Bleicken *et al*, 2014; Flores‐Romero *et al*, 2017). Although the structural organization of BAX dimers in the membrane remains controversial (Westphal *et al*, 2014; Mandal *et al*, 2016; Cosentino & Garcia‐Saez, 2017; Bleicken *et al*, 2018), some of the models proposed provide an explanation for how BAX/BAK may generate the membrane stress required for pore opening and alleviate the line tension for pore stabilization (Bleicken *et al*, 2014; Mandal *et al*, 2016; Fig 2B). Partial opening and insertion of the hairpin of helices α5–α6 in BAX/BAK dimers within the lipid headgroup region of the cytosolic leaflet of the MOM would initially generate positive curvature and membrane stress leading to pore opening (Czabotar *et al*, 2013; Bleicken *et al*, 2014). The crescent‐like shape of these two α‐helices in the context of the pore rim would then act as a scaffolding chaperone that stabilizes the high lipid curvature, decreases the line tension, and maintains the open pore state (Mandal *et al*, 2016; Bleicken *et al*, 2018; Fig 2B). The formation of high‐order oligomers with such membrane disposition would potentiate the stabilizing effect (Subburaj *et al*, 2015).

In agreement with this, atomic force microscopy (AFM) experiments on supported bilayers, electron microscopy (EM) assays in outer membrane vesicles (OMVs) and in lipid nanodiscs containing BAX confirmed protein enrichment at the rim of membrane pores of variable sizes. Remarkably, the pore wall was not completely covered by protein molecules (Xu *et al*, 2013; Subburaj *et al*, 2015; Kuwana *et al*, 2016; Salvador‐Gallego *et al*, 2016) (Fig 2C). This evidence supports a model for BAX/BAK‐mediated MOMP where oligomerization at the MOM induces the formation of heterogeneous toroidal pore structures of tunable size, which are flexible and evolve overtime leading to the release of apoptotic factors.

### BAX and BAK supramolecular structures and functions beyond MOMP

Historically, the segregation of BAX and BAK into discrete puncta at mitochondria, also known as apoptotic foci, has been linked with the apoptotic phenotype (Nechushtan *et al*, 2001; Karbowski *et al*, 2002). High‐resolution imaging techniques have recently allowed deciphering the riddles of these supramolecular structures, revealing distinct molecular architectures such as lines, arcs, and full rings (Grosse *et al*, 2016; Salvador‐Gallego *et al*, 2016). Rings and arcs of BAX similar to those found at the MOM perforated the membrane in supported lipid bilayers indicating that these higher‐order oligomerization states do not necessarily require other mitochondrial proteins (Fig 2C). BAX seems to oligomerize by subsequent addition of dimers (Subburaj *et al*, 2015), but the formation and evolution of these oligomers at the supramolecular level are vaguely understood. BAX molecules might first organize into linear and arc‐shaped structures, with some of them evolving to complete rings. Alternatively, lines and arcs might correspond to kinetically trapped assemblies in the process of pore formation. Several inter‐dimer binding surfaces have been described for BAX‐type proteins, including helices α6 and α9 (Dewson *et al*, 2009; Zhang *et al*, 2010) or the interface of helices α3/α5 (Mandal *et al*, 2016), but none of them appears to be indispensable for BAX/BAK oligomerization. Considering that BAX/BAK self‐assembly seems to be crucial for reducing line tension and pore stabilization, one could envision that the membrane itself may play a role in the organization and dynamics of BAX/BAK supramolecular arrangements by contributing to the state of minimal energy. In this scenario, the energy cost of the membrane perturbations (perhaps including initial pore opening) induced by the insertion of BAX dimers into the membrane could be reduced by the coalescence of these membrane alterations and associated BAX/BAK molecules. Such a model would explain the higher‐order assembly of BAX/BAK dimers via membrane‐mediated interactions (Harroun *et al*, 1999; Reynwar *et al*, 2007; Shlomovitz & Gov, 2009; Cowan *et al*, 2020) and provide a mechanistic basis for the pore growth observed during apoptosis (Riley *et al*, 2018; Flores‐Romero & García‐Sáez, 2020).

Connected with BAX foci, it has been proposed that BAX and BAK pores of hundreds of nanometers in diameter can also induce mitochondrial inner membrane (MIM) permeabilization and extrusion into the cytosol, leading to the release of mitochondrial DNA (mtDNA) and the activation of cGAS/STING inflammatory pathway (McArthur *et al*, 2018; Riley *et al*, 2018; Fig 2D). These discoveries have challenged our understanding of the role of BAX/BAK in apoptosis beyond that of inducing MOMP for caspase activation, which now expands to inflammation and cell‐to‐cell communication. The mechanism how BAX and BAK promote MIM poration and mtDNA release remains unknown. While some studies suggest that monomers or dimers are sufficient to form functional pores in model membranes (Kushnareva *et al*, 2012; Xu *et al*, 2013), it seems unlikely that BAX/BAK monomers or dimers would be able to induce the large membrane disruptions required for mtDNA release. One could envision a scenario in which low order oligomers of BAX/BAK induce openings at the MOM that release cytochrome c and other proteins, while large supramolecular structures may enable other cell functions including mtDNA release and inflammatory responses. The continuous growth of BAX pores upon MOMP would thereby allow a controlled release of the mitochondrial components to regulate in time these alternative functions and with them, the inflammatory outcome of apoptosis (Cosentino & Garcia‐Saez, 2018; McArthur *et al*, 2018; Riley *et al*, 2018).

However, it is important to note that additional components are present at BAX/BAK foci at mitochondria, such as Dynamin‐related protein 1 (Drp1) and Mitofusins (Karbowski *et al*, 2002; Ugarte‐Uribe & Garcia‐Saez, 2017; Ugarte‐Uribe *et al*, 2018; Hertlein *et al*, 2020), Optic atrophy‐1(OPA1)/ Metalloendopeptidase OMA1 (OMA1) (Yamaguchi *et al*, 2008; Jiang *et al*, 2014), or Voltage‐Dependent Anion Channel (VDAC) (Lauterwasser *et al*, 2016; Kim *et al*, 2019), which might contribute to this phenomenon. As MIM permeabilization appears to occur after MOMP, the driving force that makes the mitochondrial interior squeeze out through BAX/BAK macro‐pores might involve mechanical (osmotic) forces. This could possibly be due to or happen in combination with additional mitochondrial alterations like the dismantling of mitochondrial cristae. The mitochondrion‐specific lipid cardiolipin (CL), which seems to regulate the action of several BCL2 proteins (Terrones *et al*, 2004; Landeta *et al*, 2014; Bleicken *et al*, 2017; Flores‐Romero *et al*, 2019) and is also implicated in mitochondrial functions including organelle ultrastructure (Schlame & Ren, 2009), could also play a role in MOM/MIM permeabilization. Because of its unique structural properties (e.g., two negative charges, a relatively small head group and four acyl chains), CL can form highly curved inverted hexagonal structures (Grijalba *et al*, 1999; Ortiz *et al*, 1999; Unsay *et al*, 2013) and laterally segregate into defined nanodomains *in vitro* (Kawai *et al*, 2004; Sorice *et al*, 2009). These additional elements might modulate the formation, size, shape, and kinetics of BAX/BAK assemblies in apoptotic foci.

At the functional level, it is reasonable to argue that the regulation of the extent and kinetics of BAX/BAK‐induced MOMP may elicit different scenarios, which could be: (i) genomic instability and cancer, associated with partial release of cytochrome *c* and minority MOMP (Ichim *et al*, 2015), (ii) immunologically silent apoptosis, when MOMP, caspase activation and cell removal due to “eat me signals” are fast and complete (Depraetere, 2000; Singh *et al*, 2019), or (iii) immunologically active apoptosis in case of prolonged apoptotic signaling leading to mtDNA release and to activation of the cGAS/STING and perhaps Mitochondrial antiviral‐signaling protein (MAVS) pathways (Cosentino & Garcia‐Saez, 2018; McArthur *et al*, 2018; Riley *et al*, 2018; Flores‐Romero & Garcia‐Saez, 2019b). The regulation of these scenarios could be mechanistically related with the structural flexibility and dynamics of assembly of BAX/BAK structures, ranging from active monomer/dimer units to supramolecular structures with different sizes and shapes. Importantly, if the different oligomeric states of BAX and BAK exert different functions in the cell, their specific targeting could expand the application of BAX and BAK for therapy.

## Gasdermins are potent pore‐formers at the core of pyroptosis

### The GSDM family is a new class of PFPs

GSDMs represent a family of proteins that comprises six members in humans: GSDM A, B, C, D, and E (also known as DFNA5), and PJVK (also known as DFNB59), and ten in mice (GSDM A1‐3, C1‐4, D, E, and PJVK). Some of these proteins have proved to be essential for the highly inflammatory pathway of pyroptosis (Aglietti & Dueber, 2017; Galluzzi *et al*, 2018; Broz *et al*, 2020). Pyroptotic cell death is characterized by extensive cell swelling and membrane blebbing in absence of cell detachment (Chen *et al*, 2016; de Vasconcelos *et al*, 2019), which resembles the phenotype induced by PFTs during their attack to the plasma membrane of target cells (Garcia‐Saez *et al*, 2011; Ros *et al*, 2017). This, together with the essential role of GSDMs in pyroptosis, suggested that GSDMs have an intrinsic and potent pore‐forming activity that mediates osmotic lysis in pyroptosis. Accordingly, the N‐terminal domain (GSDM^NT^) of GSDMs alone displays pore‐forming activity in liposomes, which has been the basis to define GSDMs as the minimal machinery for pyroptosis execution (Aglietti *et al*, 2016; Ding *et al*, 2016; Liu *et al*, 2016).

**Figure 3:**
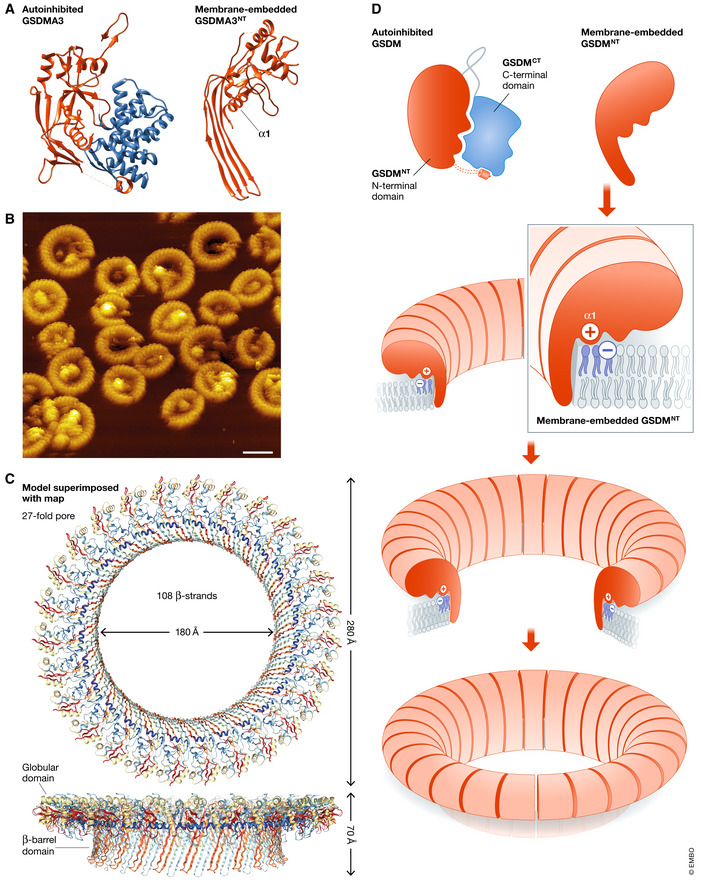
GSDMs pores evolve from toroidal to barrel structures (A) Crystal structure of GSDMA3 in its auto‐inhibited form (PDB: 5B5R). The GSDMA3^NT^ and GSDMA3^CT^ domains are colored pink and blue, respectively. Inter‐domain interactions between the GSDMA3^NT^ and the GSDMA3^CT^ keep the protein in an auto‐inhibited state (Ding *et al*, 2016). (B) GSMDs involves the form arc‐, slit, and ring‐shaped GSDMD^NT^ oligomers as imaged using time‐resolved AFM (Mulvihill *et al*, 2018). (C) Cryo‐EM structure of the GSDMA3 membrane pore (PDB: 6CB8). Atomic model of the 27‐fold symmetric GSDMA3 pore at 3.8 Å resolution (Ruan *et al*, 2018). (D) Model of pore formation by GSDMs. After cleavage, monomers of GSDM^NT^ translocate to the inner leaflet of the plasma membrane and then self‐associate into arcs or slit structures that resemble toroidal pores and later evolve into ring‐shape protein‐lined pores with a β‐barrel configuration.

Intense research during the recent years has provided insight into the structure of GSDMs (Fig 3A–D). All GSDMs (except DFNB59) display a two‐domain architecture formed by an N‐terminal (GSDM^NT^) and a C‐terminal (GSDM^CT^) domain, separated by a linker region (Fig 3A; Broz *et al*, 2020). The crystal structure of full‐length GSDMA3 (Ding *et al*, 2016) and GSDMD (Kuang *et al*, 2017) revealed that the GSDM^NT^ is inhibited by inter‐domain interactions with juxtaposed regions of the GSDM^CT^. The α‐helical fold of GSDM^CT^ interacts with the helix α1 and a short β‐hairpin located on the concave side of the β‐sheet of GSDM^NT^. Additionally, the short α‐helix at the end of the β‐sheet of GSDM^NT^ protrudes from the globular structure to interact with GSDM^CT^ (Ding *et al*, 2016). For many GSDMs, caspase‐mediated proteolytic processing induces the dissociation of the GSDM^NT^ from its auto‐inhibitory C‐domain (Fig 3A; Kuang *et al*, 2017; Liu *et al*, 2018; Liu *et al*, 2019). Free monomers of GSDM^NT^ translocate then to the inner leaflet of the plasma membrane and induce pyroptotic pores. Remarkably, the sequence and 3D‐structure of all GSDMs differ significantly from any other known PFP (Ruan *et al*, 2018; Broz *et al*, 2020). Therefore, GSDMs have emerged as a new group of PFPs with a common function in pyroptosis.

The structure of a GSDMA3^NT^ pore was recently determined by cryo‐EM (Fig 3C) (Ruan *et al*, 2018). Together with the crystal structure of full‐length GSDMA3 (Ding *et al*, 2016), these findings were the basis for a model of the transformation that this protein undergoes from the auto‐inhibited form to the active membrane‐embedded state. Cleaved GSDM^NT^ associates with negatively charged lipids at the plasma membrane via a cluster of positively charged amino acids in helix α1 (Ruan *et al*, 2018). Interaction with the lipid environment facilitates a conformational change that involves refolding into a new four‐stranded amphiphilic β‐sheet and generation of three oligomerization interfaces. The establishment of well‐defined protein‐protein interactions drives the stabilization of a ring‐shaped β‐barrel pore embedded in the membrane. The amphipathic β‐sheets are bundled together in a transmembrane configuration where the hydrophobic surfaces are in contact with the membrane core, while the hydrophilic ones are facing the pore lumen (Ding *et al*, 2016). However, the structural arrangement of the lipids in the vicinity of GSDM^NT^ pores remains unknown.

Time‐resolved AFM studies of the pores formed by human GSDMD^NT^ in model membranes provided new information about the dynamics of GSDMD assembly and pore opening (Fig 3B; Sborgi *et al*, 2016; Mulvihill *et al*, 2018). These data support a model where, instead of oligomer pre‐assembly in solution, GSDMD^NT^ monomers first bind to the membrane and then self‐associate into arcs or slit structures that later evolve into ring‐shaped pores. The formation of stable pores with a defined ring structure seems to be a robust and intrinsic property of GSDMD^NT^, as changes in protein concentration (Ding *et al*, 2016; Liu *et al*, 2016) and membrane lipid composition (Mulvihill *et al*, 2018) affect protein binding to the membrane but not pore size. Arcs and slits are also able to perforate the membrane (Mulvihill *et al*, 2018), which indicates the direct involvement of lipids in these intermediate pore structures. As discussed for BAX, protein–lipid pores can be induced when amphipathic regions of proteins bind to or insert into the membrane, lowering the energy to form a pore (Cosentino & Garcia‐Saez, 2017). In this scenario, it is reasonable to assume that the perimeter of the pore wall would be partially covered by GSDMs molecules and partially with a semi‐toroidal disposition of the membrane in the opposite side (Fig 3B and D).

### GSDMs are mechanistically similar to other β‐PFPs

GSDMs are classified as β‐PFPs based on the structural element that forms the pore wall. Compelling data obtained from orthogonal studies with different family members suggest that pores formed by GSDMs have a relatively narrow size distribution, with diameters in the order of 10–30 nm (Ding *et al*, 2016; Sborgi *et al*, 2016; Heilig *et al*, 2018; Ruan *et al*, 2018). As a result, and despite being structurally unique PFPs, GSDMs pores resemble the transmembrane β‐barrel channels formed by the membrane attack complex perforin‐like/cholesterol‐dependent cytolysin (MACPF/CDC) family (Ros & Garcia‐Saez, 2015; Gilbert, 2016). Examples of MACPF/CDC members are the bacterial cytolisins (e.g., Streptolysin O and Listeriolysin O), the complement proteins and perforin from cytotoxic T lymphocytes (Anderluh *et al*, 2014; Gilbert *et al*, 2014). Contribution of lipids to intermediate structures of different shapes is also shared between GSDMs and the MACPF/CDC families (Sonnen *et al*, 2014; Metkar *et al*, 2015; Podobnik *et al*, 2016; Mulvihill *et al*, 2018). Furthermore, although evolutionary distant, GSDMs and MACPF/CDC families have a common function in the bacterial defense systems and in immunity (Gilbert *et al*, 2013; Anderluh *et al*, 2014; Broz *et al*, 2020).

This stark similitude supports a conserved strategy of pore formation by large β‐PFPs and makes it reasonable to extrapolate some features of the MACPF/CDC pores to pyroptotic pores. Evidence obtained with different members of the MACPF/CDC family suggests that these proteins follow a mechanism of pore formation in which the rolling insertion of oligomers results in the flow of lipids from the pore rim back to the bilayer (Sonnen *et al*, 2014; Gilbert, 2016; Podobnik *et al*, 2016). In this process, lipids return from the semi‐toroidal edges in the pore to the bilayer structure, rather than being punched out into solution during oligomer insertion (Gilbert *et al*, 2013; Gilbert, 2016). Considering the dynamics of GSDMs pores, it is tempting to speculate that GSDMs could follow a similar mechanism of lipid clearance that involves the evolution of intermediate protein–lipid semi‐toroidal structures to a fully protein‐lined pore during pyroptosis (Fig 3D; Sonnen *et al*, 2014; Gilbert, 2016).

### The inflammatory pore of GSDMs

GSDMs pores are non‐selective membrane channels that mediate the secretion of intracellular components that act as activators of the immune system (Zanoni *et al*, 2016; Evavold *et al*, 2018). Chief among them, the inflammatory cytokines IL‐1β and IL‐18 can be released via these pores through non‐conventional mechanisms in a cell death‐independent manner (Heilig *et al*, 2018). The passive transport of molecules through GSDMs pores also leads to osmotic imbalance, cell bursting, and final death, which allows the unspecific release of larger damage‐associated molecular patterns (DAMPS) from cells (Broz *et al*, 2020; Ros *et al*, 2020). One interesting aspect that remains a matter of debate is whether GSDMs pores could impact the events that anticipate cell death by regulating the size of the molecules that are released from pyroptotic cells. To play a significant role in controlling contents efflux, GSDMs pores should precede cell bursting with a sufficiently long lag time, which may be regulated by opposing cellular processes. Indeed, the endosomal sorting complex required for transport (ESCRT) machinery has been shown to counterbalance GSDMD^NT^ pores during pyroptosis (Ruhl *et al*, 2018). This and other membrane repair mechanisms may, as a result, be relevant not only for protecting against cell death but also for regulating pore number and cell death dynamics. In this context, pore formation by GSDMs would emerge as a mechanism to tightly control the kinetics and extent of release of inflammatory molecules from pyroptotic cells.

## Staying open‐minded: How does MLKL execute necroptosis?

### MLKL is not similar to any other PFP

MLKL is the only effector exclusively implicated in necroptosis and the most downstream component of the pathway known so far (Galluzzi *et al*, 2018; Espiritu *et al*, 2019; Flores‐Romero *et al*, 2020). It is a cytosolic, monomeric pseudo‐kinase with no day‐job in healthy cells known to date, although evidence suggests that it may be implicated in membrane trafficking (Yoon *et al*, 2017). During necroptosis, it becomes activated by phosphorylation by receptor‐interacting protein 3 (RIP3). MLKL activation triggers its oligomerization and association with the inner leaflet of the plasma membrane, which eventually leads to membrane permeabilization and cell death (Wallach *et al*, 2016; Petrie *et al*, 2017; Petrie *et al*, 2018). Despite its central role in necroptosis, MLKL is a very intriguing protein and the mechanism how it induces plasma membrane permeabilization remains a topic of debate.

**Figure 4:**
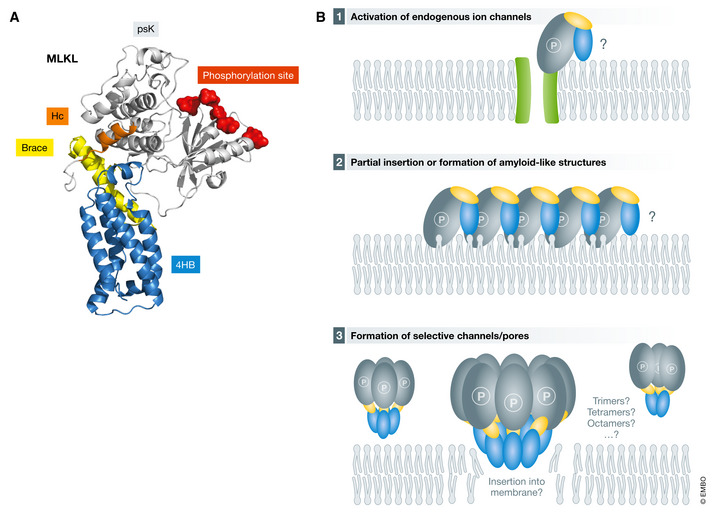
MLKL induces pores by a still unclear mechanism (A) Crystal structure of mouse MLKL (PDB: 4BTF). The 4HB domain, the brace region, and the psK domain are colored blue, orange, and gray, respectively. (B) Alternative models proposed for the mechanism how MLKL mediates plasma membrane permeabilization. (1) indirect mechanism via activation of endogenous ion channels, (2) partial insertion into the lipid bilayer, or (3) formation of defined channels or pores.

The 3D structure of inactive MLKL is comprised by two domains: an N‐terminal four helix bundle domain (4HB) and a C‐terminal pseudo‐kinase domain (psK) bridged by a flexible brace region (Murphy *et al*, 2013; Su *et al*, 2014; Fig 4A). Intensive research over the last years has demonstrated that the 4HB of MLKL acts as the killer domain, whereas the psK domain and the brace region are critical to keep in check the cell death‐inducing capacity of MLKL (Hildebrand *et al*, 2014; Su *et al*, 2014; Arnez *et al*, 2015; Tanzer *et al*, 2016). The 4HB domain of MLKL is arranged into a coiled‐coil of amphipathic α‐helices in which the hydrophobic faces are hidden in the core of the structure (Murphy *et al*, 2013; Su *et al*, 2014). This configuration has 3D homology with the HeLo‐like domain found in fungal Heterokaryon incompatibility protein S (HET‐S), which form pores by a mechanism involving the insertion of their N‐terminal α‐helix in the membrane (Daskalov *et al*, 2016).

Upon activation, MLKL is thought to undergo a conformational change that facilitates its weak interaction with PIPs at the plasma membrane through a cluster of positively charged residues located in the 4HB (Dondelinger *et al*, 2014). High‐affinity sites are then exposed, which stabilizes stronger interactions with PIPs via a rolling‐over mechanism (Quarato *et al*, 2016). Besides this, very little is known about the assembly pathway and oligomeric state of MLKL at plasma membrane. Different studies have proposed that MLKL can form trimers, tetramers, hexamers, and even higher‐order oligomers (Cai *et al*, 2014; Chen *et al*, 2014; Huang *et al*, 2017; Liu *et al*, 2017; Petrie *et al*, 2018). It is reasonable to speculate that the different reported oligomers could be intermediate states of higher‐order complexes.

The supramolecular structure of membrane MLKL oligomers promoting membrane permeabilization remains undefined too. Early studies proposed that MLKL activates endogenous ion channels, which would be the actual responsible for cell death (Cai *et al*, 2014; Chen *et al*, 2014). However, this model does not seem to hold true in light of more recent data showing that specific ion channels are not essential for necroptosis (Chen *et al*, 2014; Xia *et al*, 2016; Ousingsawat *et al*, 2017). A more general hypothesis is that MLKL directly mediates the permeabilization of the plasma membrane either as a result of its partial insertion into the lipid bilayer (Su *et al*, 2014) or by forming defined channels or pores (Fig 4B; Xia *et al*, 2016). The evidence that membrane nanopores represent a core mechanism in necroptosis supports a possible pore‐forming function for MLKL (Ros *et al*, 2017). However, whether MLKL is directly or indirectly forming necroptotic pores, alone or together with other cellular components, is something that still remains to be elucidated.

### Alternative models for pore formation by MLKL

**Figure 5:**
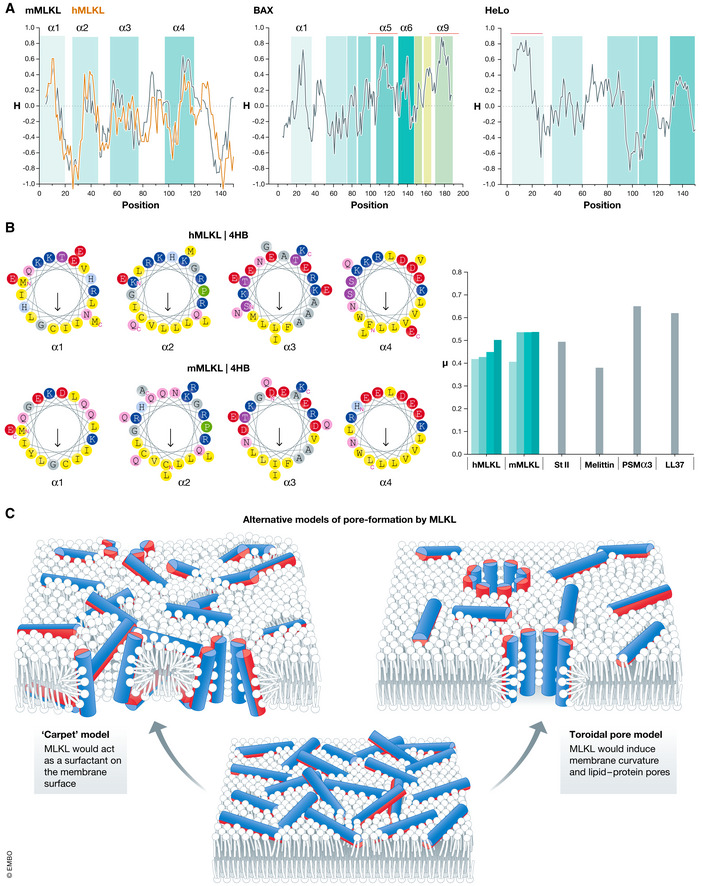
Alternative models of pore formation by the amphipathic α‐helices of MLKL (A) Hydropathy profiles of the 4HB of MLKL, the HeLo‐like N domain‐containing protein from *Chaetomium globosum* and BAX. Profiles were built with the program Protscale (https://web.expasy.org/protscale/), using the Eisenberg scale with a window of 9 amino acid residues. Segments above 0 are predicted as hydrophobic and below 0 as hydrophilic. Predicted transmembrane segments are highlighted in red. Predictions were made using two different softwares: TMHMM (http://www.cbs.dtu.dk/services/TMHMM) and TCDB (http://www.tcdb.org/progs/?tool=hydro). (B) Amphipathic nature of the α‐helices of the 4HB of MLKL. Left: Wheel projections of the α‐helices of the 4HB of human (top) or mouse (bottom) MLKL. Arrows point toward the hydrophobic face of the α‐helices. Projections were built using the server Heliquest (https://heliquest.ipmc.cnrs.fr/). Right: μ of the amphipathic α‐helices of the 4HB of MLKL, the pore‐forming domain of the α‐PFT sticholysin II and the lytic peptides melittin, LL37 and PSMα3. (C) Amphipathic α‐helices of MLKL could alter membrane integrity by two alterative models. In a carpet model, MLKL would act as a surfactant on the membrane surface, while in the toroidal pore, MLKL would induce membrane curvature and lipid–protein pores. Cylinders indicate individual amphipathic helices of the 4HB of MLKL. Hydrophobic surfaces are depicted in red and hydrophilic in blue.

One distinctive feature of pore‐forming domains is the presence of a highly hydrophobic stretch that helps partitioning into the hydrophobic core of the membrane. Amphipathic α‐helices can also function as pore‐forming domains since they are ideal for binding to membrane interfaces (Ros & Garcia‐Saez, 2015; Grage *et al*, 2016). To predict potential pore‐forming segments in MLKL, we built the hydropathy profile of the 4HB (Fig 5A). We found that none of the α‐helices in the 4HB of MLKL is sufficiently hydrophobic to be predicted to insert in membranes. This is in contrast to other α‐PFPs, including BAX or the HeLo‐like proteins, which questions the pore‐forming activity of MLKL. We also calculated the mean hydrophobic moment (µ) as a quantitative indicator of amphipathic nature of the individual helices in the 4HB (Fig 5B). The values obtained were comparable to those of single‐helix pore‐forming domains of other PFPs analyzed, although in MLKL, the hydrophobic patches of the four α‐helices cooperate in the assembly of the helical bundle fold and may thus not necessarily behave as pore‐forming domains.

Unfortunately, we still lack the structure and topology of MLKL in membranes. It remains to be disclosed whether MLKL activation facilitates the unfolding of the 4HB and the exposure of normally hidden hydrophobic surfaces. This could lead to formation a protein‐lined transmembrane pore in which the polar surfaces of the amphipathic α‐helices face the aqueous lumen and the hydrophobic sides are in contact with the membrane core. Alternatively, the amphipathic α‐helices of the 4HB might remain lying at the membrane interface region without spanning the bilayer, as it has been proposed (Fig 5C) (Su *et al*, 2014; Petrie *et al*, 2017). In such scenario, MLKL could mediate pore formation via alternative mechanisms:

1. One possibility could be that the 4HB helices of MLKL behave as surfactants and create a carpet on the membrane surface(Fig 5C) , thereby destabilizing the bilayer structure and permeabilizing the membrane once a concentration threshold at the membrane is reached, like in the case of detergents or in form of amyloid fibers (Liu *et al*, 2017). In this regard, MLKL could follow a mechanism similar to the antimicrobial peptide LL37 or the bacterial cytotoxic PSMα3 peptide, where amphipathic α‐helices are densely packed forming amyloid fibers that induce membrane destabilization (Tayeb‐Fligelman *et al*, 2017; preprint: Engelberg & Landau, 2020).
2. MLKL may act instead as an α‐PFP by inducing membrane curvature without fully spanning the hydrophobic core of the membrane (Fig 5C). A similar mechanism has been proposed for different lytic peptides and proteins such as melittin and magainin (Sengupta *et al*, 2008; Lee *et al*, 2011) and actinoporins (Alvarez *et al*, 2003; Mesa‐Galloso *et al*, 2019; Fig 5C). In this case, the asymmetric attack of the amphipathic α‐helices of the unfolded 4HB would induce membrane thinning and tension, inducing membrane curvature and toroidal pore formation (Chen *et al*, 2003; Grage *et al*, 2016; Woo & Lee, 2017). Curvature induction could be relevant not only for the membrane permeabilizing activity of MLKL but also for potential alternative functions in endosome/exosome generation (Yoon *et al*, 2017; Fan *et al*, 2019).
3. Another option would be that MLKL induces membrane curvature and permeabilization due to local high concentration together with PIPs. In this case, MLKL would behave similarly to the poorly understood fibroblast growth factor (FGF2), a mediator of the unconventional secretory pathway (Steringer *et al*, 2012; Dimou *et al*, 2019) and the HIV‐1 transactivator of transcription (HIV‐Tat) (Zeitler *et al*, 2015).

In all these pore models, the alteration of the bilayer structure by MLKL could facilitate the flip‐flop movement of lipids via a scramblase activity, thereby providing an explanation for the exposure of phosphatidylserine (PS) to the outer leaflet of the plasma membrane in necroptotic cells (Gong *et al*, 2017a,b).

### Is MLKL an intrinsic killer?

At a single cell level, necroptosis is a slow process in which cells round and detach with increasing osmotic pressure (Chen *et al*, 2016; Ros *et al*, 2017). Recent studies have shown that during the early phase of necroptosis, phosphorylated MLKL remains at the plasma membrane for a long time before cell death (Tanzer *et al*, 2015; Fan *et al*, 2019). These features contrast GSDMs and other PFPs, suggesting that MLKL may not have a potent membrane permeabilizing activity.

A question that remains open is whether this weak activity is an intrinsic property of MLKL or the result of tight regulation by the cell. Different evidence indicates that maintaining MLKL levels at the plasma membrane below a threshold prevents necroptosis (Gong *et al*, 2017b; Hildebrand *et al*, 2020; Petrie *et al*, 2020; Samson *et al*, 2020). Besides RIP3, several proteins and co‐factors have been identified as modulators of MLKL activity in cells. This includes the TAM kinases (Najafov *et al*, 2019), the heat shock protein 90 (HSP90) (Bigenzahn *et al*, 2016; Jacobsen *et al*, 2016), ATP (Petrie *et al*, 2018), and the highly phosphorylated versions of soluble inositol phosphates (i.e., IP4, IP5, and IP6) (Dovey *et al*, 2018; McNamara *et al*, 2019). Moreover, membrane repair mechanisms involving exosome release, endocytosis, and exocytosis play a counteracting role in necroptosis (Yoon *et al*, 2017; Gong *et al*, 2017b; Fan *et al*, 2019). The fact that MLKL orthologues from different species are not exchangeable among different host cells also supports the hypothesis that host‐specific factors might regulate MLKL activity in cells (Tanzer *et al*, 2016; Petrie *et al*, 2018).

During the commitment phase of necroptosis, MLKL mediates a number of cellular alterations including PS exposure, intracellular vesicle trafficking, exosome release and production of inflammatory cytokines, and DAMPs (Yoon *et al*, 2017; Gong *et al*, 2017a,b; Douanne *et al*, 2019; Espiritu *et al*, 2019). Given the multiple processes activated downstream of MLKL translocation to the plasma membrane, an interesting possibility would be that MLKL is not an intrinsic killer or that its weak activity is functionally relevant. In this scenario, the primary role of MLKL could be to promote these distinctive intracellular modifications in order to allow necroptotic cells to communicate in a unique manner with the environment. Whether the result of MLKL activation is cell death would then depend on the extension of MLKL‐dependent membrane damage and the efficiency of the cellular mechanisms that promote or counterbalance MLKL assembly at the plasma membrane.

## Pores by BAX/BAK, GSDMs, and MLKL: the devil is in the detail

BAX/BAK, GSDMs, and MLKL have evolved to have a common role as executors of regulated cell death via membrane permeabilization. There are similarities in the general mechanisms of action of these proteins. With the exception of BAK, they are all produced as soluble proteins that undergo extensive conformational changes upon activation and interaction with the membrane. Membrane binding is driven, at least partially, by electrostatic interactions of positively charged residues located on the protein surface with anionic lipids in the target membranes. Hydrophobic interactions between the bilayer and amphipathic and/or transmembrane segments of these proteins also contribute to membrane partition and insertion. Once at the membrane, all of them form oligomers that are able to disrupt the bilayer, allowing the passive flux of molecules.

However, BAX/BAK, GSDMs, and MLKL strongly differ in the way they alter the structure and permeability of cellular membranes. This is reasonable, as distinct types of effectors may be required to permeabilize target membranes with different protein and lipid composition, as well as mechanical properties. To execute apoptosis, BAX is targeted to the MOM, while GSDMs and MLKL bind to the inner leaflet of the plasma membrane to mediate pyroptosis and necroptosis. The ability of BAX/BAK to open protein–lipid pores is very sensitive to the membrane lipid composition (Basanez *et al*, 2002; Terrones *et al*, 2004), which may be a regulatory mechanism during apoptosis. On the other hand, GSDMs and MLKL target the plasma membrane by a common mechanism of interaction with anionic PIPs (Parisi *et al*, 2018; Ros *et al*, 2020). These two proteins can also be found in organelles. MLKL binds to endosomes and regulates intracellular vesicle trafficking and receptor recycling (Yoon *et al*, 2017). Some studies have suggested that GSDMs can disrupt mitochondria via its ability to recognize CL (Ding *et al*, 2016; Huang *et al*, 2020). However, it remains unclear how GSDMs and MLKL discriminate between different organelles and which are the conditions that trigger their specific targeting to different cellular membranes.

Based on the secondary structure of the pores they form, BAX/BAK, and perhaps MLKL, are classified as α‐PFPs, while GSDMs are β‐PFPs. These structural differences strongly determine the molecular properties of the membrane pores built by these proteins. α‐helical proteins tend to form more flexible pores in which oligomeric structures are weakly associated with intra‐chain hydrogen bonds interactions. In contrast, β‐barrel pores present high stability by inter‐strand hydrogen bonds formation between juxtaposed chains (Gilbert *et al*, 2014; Ros & Garcia‐Saez, 2015). These differences would explain why GSDMs have well‐defined protein–protein interfaces that stabilize the β‐barrel arrangement in the membrane and are amenable for structural studies. Instead, BAX and BAK assemblies are less rigid and more heterogeneous in terms of interaction interfaces and supramolecular structures, and have escaped so far high‐resolution structural determination. It will be interesting to learn how the structure of MLKL oligomers in the membrane compares to that of BAX/BAK and GSDMs.

Along these lines, while a common feature of the mechanism of BAX/BAK and GSDMs is the formation of arc‐shaped structures that only partially cover the pore rim, the intramolecular interactions that stabilize these protein–lipid assemblies are different. In the case of GSDMs, it seems that arcs are intermediates transitioning into complete rings in which lipids are excluded. However, the BAX/BAK pores are flexible and dynamic, and their size depends on protein density at the membrane and on lipid composition (Landeta *et al*, 2011; Bleicken *et al*, 2013; Landeta *et al*, 2015; Subburaj *et al*, 2015; Salvador‐Gallego *et al*, 2016; Cosentino & Garcia‐Saez, 2017). This, together with the difficulty to define interaction surfaces between BAX/BAK dimers, supports a model in which protein dimers and lipids could be intermixed with each other also in the ring‐like structures. Further research is needed to confirm this distinctive property of BAX pores compared to GSDMs ones.

## Why so many ways of piercing membranes to kill a cell?

Altogether, the different mechanisms of pore formation are likely to define the signaling processes activated downstream of membrane permeabilization in all forms of regulated cell death. On the one hand, the MOM is a membrane naturally permeable to molecules up to 5 kDa due to the presence of several porins (e.g., VDACs) and a singular lipid composition (Fleischer *et al*, 1967; Vance, 1990; Kagan *et al*, 2004). In this context, the growing nature of BAX pores may be key to enable temporal control of the release of mitochondrial contents ranging from small proteins like cytochrome c, to intermediate molecules like SMAC and to larger polymers like mtDNA (Kuwana *et al*, 2002; McArthur *et al*, 2018; Riley *et al*, 2018). The heterogeneity in BAX/BAK pores may therefore underline alternative cellular responses in apoptosis.

On the other hand, although GSDMs and MLKL target the same cellular membrane, the mechanism how they induce membrane permeabilization presents remarkable differences. In pyroptosis, GSDMD^NT^ forms big and non‐selective pores that mediate the release of proteins, like interleukins and other DAMPs, even before cell death. In contrast, MLKL induces the formation of smaller pores of limited permeability in necroptosis. These differences could set the basis for the different kinetics of death and morphological features of pyroptotic and necroptotic cells. It is tempting to speculate that in the tissue, two outcomes could be possible. On the one hand, content efflux might be transient and highly controlled with prevalence of plasma membrane repair and recovery in the absence of cell death. Alternatively, dominance of loss of ion homeostasis and osmotic swelling would lead to plasma membrane bursting and complete exposure of the cellular components upon cell death. The possibility that these two scenarios may communicate distinct signals to the microenvironment is intriguing.

## Conclusions and future perspectives

Despite decades of intense research, the structure and dynamic evolution of the apoptotic pores formed by BAX and BAK and the possible roles of other mitochondrial proteins and/or lipids remain open questions. What are the functional consequences of the structural flexibility of BAX/BAK supramolecular structures? Are there mechanistic and functional links between MOMP and MIMP? Our knowledge about necroptosis and pyroptosis is continuously expanding, but the functional impact that membrane permeabilization induced by GSDMs and MLKL is only starting to be grasped. We are still questioning whether MLKL is a PFP at all. How does the membrane repair machinery counterbalance the number and size of pores in pyroptosis and necroptosis? Is there a role for sub‐lytic activation of GSDMs and MLKL?

Pore formation has emerged as a common execution step in the signaling pathways leading to the different types of regulated cell death. By inducing or creating membrane pores of distinct nature, BAX/BAK, GSDMs, and MLKL control the movement of molecules across membranes and determine the cell’s fate. They are also directly responsible for the type and timing of contents released and their effect in the organism. Therefore, understanding how membrane pores are regulated and assembled holds a significant potential to devise new strategies to not only control cell death, but also modulate the inflammatory and immunologic effects of these processes for therapy.

## References

[embj2020105753-bib-0001] Aglietti RA , Estevez A , Gupta A , Ramirez MG , Liu PS , Kayagaki N , Ciferri C , Dixit VM , Dueber EC (2016) GsdmD p30 elicited by caspase‐11 during pyroptosis forms pores in membranes. Proc Natl Acad Sci USA 113: 7858–7863 27339137 10.1073/pnas.1607769113 PMC4948338

[embj2020105753-bib-0002] Aglietti RA , Dueber EC (2017) Recent insights into the molecular mechanisms underlying pyroptosis and gasdermin family functions. Trends Immunol 38: 261–271 28196749 10.1016/j.it.2017.01.003

[embj2020105753-bib-0003] Alvarez C , Casallanovo F , Shida CS , Nogueira LV , Martinez D , Tejuca M , Pazos IF , Lanio ME , Menestrina G , Lissi E *et al* (2003) Binding of sea anemone pore‐forming toxins sticholysins I and II to interfaces–modulation of conformation and activity, and lipid‐protein interaction. Chem Phys Lipids 122: 97–105 12598041 10.1016/s0009-3084(02)00181-0

[embj2020105753-bib-0004] Anderluh G , Kisovec M , Krasevec N , Gilbert RJ (2014) Distribution of MACPF/CDC proteins. Subcell Biochem 80: 7–30 24798005 10.1007/978-94-017-8881-6_2

[embj2020105753-bib-0005] Arnez KH , Kindlova M , Bokil NJ , Murphy JM , Sweet MJ , Guncar G (2015) Analysis of the N‐terminal region of human MLKL, as well as two distinct MLKL isoforms, reveals new insights into necroptotic cell death. Biosci Rep 36: e00291 26704887 10.1042/BSR20150246 PMC4725247

[embj2020105753-bib-0006] Basanez G , Nechushtan A , Drozhinin O , Chanturiya A , Choe E , Tutt S , Wood KA , Hsu Y , Zimmerberg J , Youle RJ (1999) Bax, but not Bcl‐xL, decreases the lifetime of planar phospholipid bilayer membranes at subnanomolar concentrations. Proc Natl Acad Sci USA 96: 5492–5497 10318911 10.1073/pnas.96.10.5492 PMC21887

[embj2020105753-bib-0007] Basanez G , Sharpe JC , Galanis J , Brandt TB , Hardwick JM , Zimmerberg J (2002) Bax‐type apoptotic proteins porate pure lipid bilayers through a mechanism sensitive to intrinsic monolayer curvature. J Biol Chem 277: 49360–49365 12381734 10.1074/jbc.M206069200

[embj2020105753-bib-0008] Basanez G , Soane L , Hardwick JM (2012) A new view of the lethal apoptotic pore. PLoS Biol 10: e1001399 23049484 10.1371/journal.pbio.1001399 PMC3457931

[embj2020105753-bib-0009] Bigenzahn JW , Fauster A , Rebsamen M , Kandasamy RK , Scorzoni S , Vladimer GI , Muller AC , Gstaiger M , Zuber J , Bennett KL *et al* (2016) An inducible retroviral expression system for tandem affinity purification mass‐spectrometry‐based proteomics identifies mixed lineage kinase domain‐like protein (MLKL) as an heat shock protein 90 (HSP90) client. Mol Cell Proteomics 15: 1139–1150 26933192 10.1074/mcp.O115.055350 PMC4813694

[embj2020105753-bib-0010] Bleicken S , Landeta O , Landajuela A , Basanez G , Garcia‐Saez AJ (2013) Proapoptotic Bax and Bak proteins form stable protein‐permeable pores of tunable size. J Biol Chem 288: 33241–33252 24100034 10.1074/jbc.M113.512087 PMC3829170

[embj2020105753-bib-0011] Bleicken S , Jeschke G , Stegmueller C , Salvador‐Gallego R , Garcia‐Saez AJ , Bordignon E (2014) Structural model of active Bax at the membrane. Mol Cell 56: 496–505 25458844 10.1016/j.molcel.2014.09.022 PMC4869853

[embj2020105753-bib-0012] Bleicken S , Hantusch A , Das KK , Frickey T , Garcia‐Saez AJ (2017) Quantitative interactome of a membrane Bcl‐2 network identifies a hierarchy of complexes for apoptosis regulation. Nat Commun 8: 73 28706229 10.1038/s41467-017-00086-6 PMC5509671

[embj2020105753-bib-0013] Bleicken S , Assafa TE , Stegmueller C , Wittig A , Garcia‐Saez AJ , Bordignon E (2018) Topology of active, membrane‐embedded Bax in the context of a toroidal pore. Cell Death Differ 25: 1717–1731 30185826 10.1038/s41418-018-0184-6 PMC6180131

[embj2020105753-bib-0014] Broz P , Pelegrin P , Shao F (2020) The gasdermins, a protein family executing cell death and inflammation. Nat Rev Immunol 20: 143–157 31690840 10.1038/s41577-019-0228-2

[embj2020105753-bib-0015] Cai Z , Jitkaew S , Zhao J , Chiang HC , Choksi S , Liu J , Ward Y , Wu LG , Liu ZG (2014) Plasma membrane translocation of trimerized MLKL protein is required for TNF‐induced necroptosis. Nat Cell Biol 16: 55–65 24316671 10.1038/ncb2883 PMC8369836

[embj2020105753-bib-0016] Chen FY , Lee MT , Huang HW (2003) Evidence for membrane thinning effect as the mechanism for peptide‐induced pore formation. Biophys J 84: 3751–3758 12770881 10.1016/S0006-3495(03)75103-0 PMC1302957

[embj2020105753-bib-0017] Chen X , Li W , Ren J , Huang D , He WT , Song Y , Yang C , Li W , Zheng X , Chen P *et al* (2014) Translocation of mixed lineage kinase domain‐like protein to plasma membrane leads to necrotic cell death. Cell Res 24: 105–121 24366341 10.1038/cr.2013.171 PMC3879712

[embj2020105753-bib-0018] Chen X , He WT , Hu L , Li J , Fang Y , Wang X , Xu X , Wang Z , Huang K , Han J (2016) Pyroptosis is driven by non‐selective gasdermin‐D pore and its morphology is different from MLKL channel‐mediated necroptosis. Cell Res 26: 1007–1020 27573174 10.1038/cr.2016.100 PMC5034106

[embj2020105753-bib-0019] Cosentino K , Ros U , Garcia‐Saez AJ (2016) Assembling the puzzle: oligomerization of alpha‐pore forming proteins in membranes. Biochem Biophys Acta 1858: 457–466 26375417 10.1016/j.bbamem.2015.09.013 PMC4869852

[embj2020105753-bib-0020] Cosentino K , Garcia‐Saez AJ (2017) Bax and Bak pores: are we closing the circle? Trends Cell Biol 27: 266–275 27932064 10.1016/j.tcb.2016.11.004 PMC5898608

[embj2020105753-bib-0021] Cosentino K , Garcia‐Saez AJ (2018) MIM through MOM: the awakening of Bax and Bak pores. EMBO J 37: e100340. 30135068 10.15252/embj.2018100340 PMC6120657

[embj2020105753-bib-0022] Cowan AD , Smith NA , Sandow JJ , Kapp EA , Rustam YH , Murphy JM , Brouwer JM , Bernardini JP , Roy MJ , Wardak AZ *et al* (2020) BAK core dimers bind lipids and can be bridged by them. Nat Struct Mol Biol 10.1038/s41594-020-0494-5 32929280

[embj2020105753-bib-0023] Czabotar PE , Westphal D , Dewson G , Ma S , Hockings C , Fairlie WD , Lee EF , Yao S , Robin AY , Smith BJ *et al* (2013) Bax crystal structures reveal how BH3 domains activate Bax and nucleate its oligomerization to induce apoptosis. Cell 152: 519–531 23374347 10.1016/j.cell.2012.12.031

[embj2020105753-bib-0024] Dal Peraro M , van der Goot FG (2016) Pore‐forming toxins: ancient, but never really out of fashion. Nat Rev Microbiol 14: 77–92 26639780 10.1038/nrmicro.2015.3

[embj2020105753-bib-0025] Daskalov A , Habenstein B , Sabate R , Berbon M , Martinez D , Chaignepain S , Coulary‐Salin B , Hofmann K , Loquet A , Saupe SJ (2016) Identification of a novel cell death‐inducing domain reveals that fungal amyloid‐controlled programmed cell death is related to necroptosis. Proc Natl Acad Sci USA 113: 2720–2725 26903619 10.1073/pnas.1522361113 PMC4790977

[embj2020105753-bib-0026] de Vasconcelos NM , Van Opdenbosch N , Van Gorp H , Parthoens E , Lamkanfi M (2019) Single‐cell analysis of pyroptosis dynamics reveals conserved GSDMD‐mediated subcellular events that precede plasma membrane rupture. Cell Death Differ 26: 146–161 29666477 10.1038/s41418-018-0106-7 PMC6294780

[embj2020105753-bib-0027] Deamer D (2016) Membranes and the origin of life: a century of conjecture. J Mol Evol 83: 159–168 27913841 10.1007/s00239-016-9770-8

[embj2020105753-bib-0028] Depraetere V (2000) "Eat me" signals of apoptotic bodies. Nat Cell Biol 2: E104 10854338 10.1038/35014098

[embj2020105753-bib-0029] Dewson G , Kratina T , Sim HW , Puthalakath H , Adams JM , Colman PM , Kluck RM (2008) To trigger apoptosis, Bak exposes its BH3 domain and homodimerizes via BH3:groove interactions. Mol Cell 30: 369–380 18471982 10.1016/j.molcel.2008.04.005

[embj2020105753-bib-0030] Dewson G , Kratina T , Czabotar P , Day CL , Adams JM , Kluck RM (2009) Bak activation for apoptosis involves oligomerization of dimers via their alpha6 helices. Mol Cell 36: 696–703 19941828 10.1016/j.molcel.2009.11.008

[embj2020105753-bib-0031] Dewson G , Ma S , Frederick P , Hockings C , Tan I , Kratina T , Kluck RM (2012) Bax dimerizes via a symmetric BH3:groove interface during apoptosis. Cell Death Differ 19: 661–670 22015607 10.1038/cdd.2011.138 PMC3307980

[embj2020105753-bib-0032] Dimou E , Cosentino K , Platonova E , Ros U , Sadeghi M , Kashyap P , Katsinelos T , Wegehingel S , Noe F , Garcia‐Saez AJ *et al* (2019) Single event visualization of unconventional secretion of FGF2. J Cell Biol 218: 683–699 30470711 10.1083/jcb.201802008 PMC6363455

[embj2020105753-bib-0033] Ding J , Wang K , Liu W , She Y , Sun Q , Shi J , Sun H , Wang DC , Shao F (2016) Pore‐forming activity and structural autoinhibition of the gasdermin family. Nature 535: 111–116 27281216 10.1038/nature18590

[embj2020105753-bib-0034] Dondelinger Y , Declercq W , Montessuit S , Roelandt R , Goncalves A , Bruggeman I , Hulpiau P , Weber K , Sehon CA , Marquis RW *et al* (2014) MLKL compromises plasma membrane integrity by binding to phosphatidylinositol phosphates. Cell Rep 7: 971–981 24813885 10.1016/j.celrep.2014.04.026

[embj2020105753-bib-0035] Douanne T , Andre‐Gregoire G , Trillet K , Thys A , Papin A , Feyeux M , Hulin P , Chiron D , Gavard J , Bidere N (2019) Pannexin‐1 limits the production of proinflammatory cytokines during necroptosis. EMBO Rep 20: e47840 31410978 10.15252/embr.201947840 PMC6776911

[embj2020105753-bib-0036] Dovey CM , Diep J , Clarke BP , Hale AT , McNamara DE , Guo H , Brown Jr NW , Cao JY , Grace CR , Gough PJ *et al* (2018) MLKL requires the inositol phosphate code to execute necroptosis. Mol Cell 70: 936–948.e7 29883610 10.1016/j.molcel.2018.05.010 PMC5994928

[embj2020105753-bib-0037] Edlich F , Banerjee S , Suzuki M , Cleland MM , Arnoult D , Wang CX , Neutzner A , Tjandra N , Youle RJ (2011) Bcl‐x(L) Retrotranslocates Bax from the mitochondria into the cytosol. Cell 145: 104–116 21458670 10.1016/j.cell.2011.02.034 PMC3070914

[embj2020105753-bib-0038] Engelberg Y , Landau M (2020) The human LL‐37(17–29) antimicrobial peptide reveals a functional supramolecular nanostructure. bioRxiv 10.1101/2020.02.04.933432 [PREPRINT] PMC7403366 32753597

[embj2020105753-bib-0039] Epand RF , Martinou JC , Montessuit S , Epand RM (2003) Transbilayer lipid diffusion promoted by Bax: implications for apoptosis. Biochemistry 42: 14576–14582 14661970 10.1021/bi035348w

[embj2020105753-bib-0040] Espiritu RA , Pedrera L , Ros U (2019) Chapter Seven – Tuning the way to die: implications of membrane perturbations in necroptosis, In Advances in biomembranes and lipid self‐assembly, Iglič A, Rappolt M, García‐Sáez AJ (eds), pp 201–247. Cambridge, MA: Academic Press

[embj2020105753-bib-0041] Evavold CL , Ruan J , Tan Y , Xia S , Wu H , Kagan JC (2018) The pore‐forming protein gasdermin D regulates interleukin‐1 secretion from living macrophages. Immunity 48: 35–44.e36 29195811 10.1016/j.immuni.2017.11.013 PMC5773350

[embj2020105753-bib-0042] Fan W , Guo J , Gao B , Zhang W , Ling L , Xu T , Pan C , Li L , Chen S , Wang H *et al* (2019) Flotillin‐mediated endocytosis and ALIX‐syntenin‐1‐mediated exocytosis protect the cell membrane from damage caused by necroptosis. Sci Signal 12: eaaw3423 31138766 10.1126/scisignal.aaw3423

[embj2020105753-bib-0043] Fleischer S , Rouser G , Fleischer B , Casu A , Kritchevsky G (1967) Lipid composition of mitochondria from bovine heart, liver, and kidney. J Lipid Res 8: 170–180 4292227

[embj2020105753-bib-0044] Flores‐Romero H , Garcia‐Porras M , Basanez G (2017) Membrane insertion of the BAX core, but not latch domain, drives apoptotic pore formation. Sci Rep 7: 16259 29176554 10.1038/s41598-017-16384-4 PMC5701199

[embj2020105753-bib-0045] Flores‐Romero H , Garcia‐Saez AJ (2019a) The incomplete puzzle of the BCL2 proteins. Cells 8: 1176 10.3390/cells8101176 PMC6830314 31569576

[embj2020105753-bib-0046] Flores‐Romero H , Garcia‐Saez AJ (2019b) MAVS‐induced mitochondrial membrane remodeling. FEBS J 286: 1540–1542 30957952 10.1111/febs.14822

[embj2020105753-bib-0047] Flores‐Romero H , Landeta O , Ugarte‐Uribe B , Cosentino K , Garcia‐Porras M , Garcia‐Saez AJ , Basanez G (2019) BFL1 modulates apoptosis at the membrane level through a bifunctional and multimodal mechanism showing key differences with BCLXL. Cell Death Differ 26: 1880–1894 30560933 10.1038/s41418-018-0258-5 PMC6748131

[embj2020105753-bib-0048] Flores‐Romero H , Ros U , García‐Sáez AJ (2020) A lipid perspective on regulated cell death In International review of cell and molecular biology, Galluzzi L, Spetz J (eds), Vol. 351, pp. 197–236. Cambridge, MA: Academic Press 10.1016/bs.ircmb.2019.11.004 32247580

[embj2020105753-bib-0049] Flores‐Romero H , García‐Sáez AJ (2020) Lipids glue BAK dimers together. Nat Struct Mol Biol 10.1038/s41594-020-00516-y 32994578

[embj2020105753-bib-0050] Fuertes G , Garcia‐Saez AJ , Esteban‐Martin S , Gimenez D , Sanchez‐Munoz OL , Schwille P , Salgado J (2010a) Pores formed by Baxalpha5 relax to a smaller size and keep at equilibrium. Biophys J 99: 2917–2925 21044589 10.1016/j.bpj.2010.08.068 PMC2966003

[embj2020105753-bib-0051] Fuertes G , Gimenez D , Esteban‐Martin S , Garcia‐Saez A , Sanchez O , Salgado J (2010b) Role of membrane lipids for the activity of pore forming peptides and proteins. Adv Exp Med Biol 677: 31–55 20687479 10.1007/978-1-4419-6327-7_4

[embj2020105753-bib-0052] Fuertes G (2011) Baxα5 at lipid membranes: structure, assembly and pore formation. Universitat de València. Ph.D thesis

[embj2020105753-bib-0200] Fuertes G , Gimenez D , Esteban‐Martin S , Sanchez‐Munoz OL , Salgado J (2011) A lipocentric view of peptide‐induced pores. Eur Biophys J 40: 399–415 21442255 10.1007/s00249-011-0693-4 PMC3070086

[embj2020105753-bib-0053] Galluzzi L , Vitale I , Aaronson SA , Abrams JM , Adam D , Agostinis P , Alnemri ES , Altucci L , Amelio I , Andrews DW *et al* (2018) Molecular mechanisms of cell death: recommendations of the Nomenclature Committee on Cell Death 2018. Cell Death Differ 25: 486–541 29362479 10.1038/s41418-017-0012-4 PMC5864239

[embj2020105753-bib-0054] Garcia‐Saez AJ , Coraiola M , Dalla Serra M , Mingarro I , Menestrina G , Salgado J (2005) Peptides derived from apoptotic Bax and Bid reproduce the poration activity of the parent full‐length proteins. Biophys J 88: 3976–3990 15778450 10.1529/biophysj.104.058008 PMC1305629

[embj2020105753-bib-0055] Garcia‐Saez AJ , Coraiola M , Serra MD , Mingarro I , Muller P , Salgado J (2006) Peptides corresponding to helices 5 and 6 of Bax can independently form large lipid pores. FEBS J 273: 971–981 16478471 10.1111/j.1742-4658.2006.05123.x

[embj2020105753-bib-0056] Garcia‐Saez AJ , Chiantia S , Salgado J , Schwille P (2007) Pore formation by a Bax‐derived peptide: effect on the line tension of the membrane probed by AFM. Biophys J 93: 103–112 17416629 10.1529/biophysj.106.100370 PMC1914428

[embj2020105753-bib-0057] Garcia‐Saez AJ , Buschhorn SB , Keller H , Anderluh G , Simons K , Schwille P (2011) Oligomerization and pore formation by equinatoxin II inhibit endocytosis and lead to plasma membrane reorganization. J Biol Chem 286: 37768–37777 21885440 10.1074/jbc.M111.281592 PMC3199519

[embj2020105753-bib-0058] Gilbert RJ , Mikelj M , Dalla Serra M , Froelich CJ , Anderluh G (2013) Effects of MACPF/CDC proteins on lipid membranes. Cell Mol Life Sci 70: 2083–2098 22983385 10.1007/s00018-012-1153-8 PMC11114033

[embj2020105753-bib-0059] Gilbert RJ , Dalla Serra M , Froelich CJ , Wallace MI , Anderluh G (2014) Membrane pore formation at protein‐lipid interfaces. Trends Biochem Sci 39: 510–516 25440714 10.1016/j.tibs.2014.09.002

[embj2020105753-bib-0060] Gilbert RJ (2016) Protein‐lipid interactions and non‐lamellar lipidic structures in membrane pore formation and membrane fusion. Biochem Biophys Acta 1858: 487–499 26654785 10.1016/j.bbamem.2015.11.026

[embj2020105753-bib-0061] Gong YN , Guy C , Crawford JC , Green DR (2017a) Biological events and molecular signaling following MLKL activation during necroptosis. Cell Cycle 16: 1748–1760 28854080 10.1080/15384101.2017.1371889 PMC5628637

[embj2020105753-bib-0062] Gong YN , Guy C , Olauson H , Becker JU , Yang M , Fitzgerald P , Linkermann A , Green DR (2017b) ESCRT‐III acts downstream of MLKL to regulate necroptotic cell death and its consequences. Cell 169: 286–300.e216 28388412 10.1016/j.cell.2017.03.020 PMC5443414

[embj2020105753-bib-0063] Grage SL , Afonin S , Kara S , Buth G , Ulrich AS (2016) Membrane thinning and thickening induced by membrane‐active amphipathic peptides. Front Cell Dev Biol 4: 65 27595096 10.3389/fcell.2016.00065 PMC4999517

[embj2020105753-bib-0064] Griffiths GJ , Corfe BM , Savory P , Leech S , Esposti MD , Hickman JA , Dive C (2001) Cellular damage signals promote sequential changes at the N‐terminus and BH‐1 domain of the pro‐apoptotic protein Bak. Oncogene 20: 7668–7676 11753644 10.1038/sj.onc.1204995

[embj2020105753-bib-0065] Grijalba MT , Vercesi AE , Schreier S (1999) Ca^2+^‐induced increased lipid packing and domain formation in submitochondrial particles. A possible early step in the mechanism of Ca^2+^‐stimulated generation of reactive oxygen species by the respiratory chain. Biochemistry 38: 13279–13287 10529202 10.1021/bi9828674

[embj2020105753-bib-0066] Grosse L , Wurm CA , Bruser C , Neumann D , Jans DC , Jakobs S (2016) Bax assembles into large ring‐like structures remodeling the mitochondrial outer membrane in apoptosis. EMBO J 35: 402–413 26783364 10.15252/embj.201592789 PMC4755111

[embj2020105753-bib-0067] Harroun TA , Heller WT , Weiss TM , Yang L , Huang HW (1999) Experimental evidence for hydrophobic matching and membrane‐mediated interactions in lipid bilayers containing gramicidin. Biophys J 76: 937–945 9929495 10.1016/S0006-3495(99)77257-7 PMC1300095

[embj2020105753-bib-0068] Heilig R , Dick MS , Sborgi L , Meunier E , Hiller S , Broz P (2018) The Gasdermin‐D pore acts as a conduit for IL‐1beta secretion in mice. Eur J Immunol 48: 584–592 29274245 10.1002/eji.201747404

[embj2020105753-bib-0069] Hertlein V , Flores‐Romero H , Das KK , Fischer S , Heunemann M , Calleja‐Felipe M , Knafo S , Hipp K , Harter K , Fitzgerald JC *et al* (2020) MERLIN: a novel BRET‐based proximity biosensor for studying mitochondria‐ER contact sites. Life Sci Alliance 3: e201900600 31818884 10.26508/lsa.201900600 PMC6910062

[embj2020105753-bib-0070] Hildebrand JM , Tanzer MC , Lucet IS , Young SN , Spall SK , Sharma P , Pierotti C , Garnier JM , Dobson RC , Webb AI *et al* (2014) Activation of the pseudokinase MLKL unleashes the four‐helix bundle domain to induce membrane localization and necroptotic cell death. Proc Natl Acad Sci USA 111: 15072–15077 25288762 10.1073/pnas.1408987111 PMC4210347

[embj2020105753-bib-0071] Hildebrand JM , Kauppi M , Majewski IJ , Liu Z , Cox AJ , Miyake S , Petrie EJ , Silk MA , Li Z , Tanzer MC *et al* (2020) A missense mutation in the MLKL brace region promotes lethal neonatal inflammation and hematopoietic dysfunction. Nat Commun 11: 3150 32561755 10.1038/s41467-020-16819-z PMC7305203

[embj2020105753-bib-0072] Huang D , Zheng X , Wang ZA , Chen X , He WT , Zhang Y , Xu JG , Zhao H , Shi W , Wang X *et al* (2017) The MLKL channel in necroptosis is an octamer formed by tetramers in a dyadic process. Mol Cell Biol 37: e00497‐16 27920255 10.1128/MCB.00497-16 PMC5311246

[embj2020105753-bib-0073] Huang LS , Hong Z , Wu W , Xiong S , Zhong M , Gao X , Rehman J , Malik AB (2020) mtDNA activates cGAS signaling and suppresses the YAP‐mediated endothelial cell proliferation program to promote inflammatory injury. Immunity 52: 475–486.e5 32164878 10.1016/j.immuni.2020.02.002 PMC7266657

[embj2020105753-bib-0074] Ichim G , Lopez J , Ahmed SU , Muthalagu N , Giampazolias E , Delgado ME , Haller M , Riley JS , Mason SM , Athineos D *et al* (2015) Limited mitochondrial permeabilization causes DNA damage and genomic instability in the absence of cell death. Mol Cell 57: 860–872 25702873 10.1016/j.molcel.2015.01.018 PMC4352766

[embj2020105753-bib-0075] Jacobsen AV , Lowes KN , Tanzer MC , Lucet IS , Hildebrand JM , Petrie EJ , van Delft MF , Liu Z , Conos SA , Zhang JG *et al* (2016) HSP90 activity is required for MLKL oligomerisation and membrane translocation and the induction of necroptotic cell death. Cell Death Dis 7: e2051 26775703 10.1038/cddis.2015.386 PMC4816171

[embj2020105753-bib-0076] Jiang X , Jiang H , Shen Z , Wang X (2014) Activation of mitochondrial protease OMA1 by Bax and Bak promotes cytochrome c release during apoptosis. Proc Natl Acad Sci USA 111: 14782–14787 25275009 10.1073/pnas.1417253111 PMC4205663

[embj2020105753-bib-0077] Kagan VE , Borisenko GG , Tyurina YY , Tyurin VA , Jiang J , Potapovich AI , Kini V , Amoscato AA , Fujii Y (2004) Oxidative lipidomics of apoptosis: redox catalytic interactions of cytochrome c with cardiolipin and phosphatidylserine. Free Radic Biol Med 37: 1963–1985 15544916 10.1016/j.freeradbiomed.2004.08.016

[embj2020105753-bib-0078] Kagan BL (2012) Membrane pores in the pathogenesis of neurodegenerative disease. Prog Mol Biol Transl Sci 107: 295–325 22482454 10.1016/B978-0-12-385883-2.00001-1

[embj2020105753-bib-0079] Karatekin E , Sandre O , Guitouni H , Borghi N , Puech PH , Brochard‐Wyart F (2003) Cascades of transient pores in giant vesicles: line tension and transport. Biophys J 84: 1734–1749 12609875 10.1016/S0006-3495(03)74981-9 PMC1302742

[embj2020105753-bib-0080] Karbowski M , Lee YJ , Gaume B , Jeong SY , Frank S , Nechushtan A , Santel A , Fuller M , Smith CL , Youle RJ (2002) Spatial and temporal association of Bax with mitochondrial fission sites, Drp1, and Mfn2 during apoptosis. J Cell Biol 159: 931–938 12499352 10.1083/jcb.200209124 PMC2173996

[embj2020105753-bib-0081] Kawai F , Shoda M , Harashima R , Sadaie Y , Hara H , Matsumoto K (2004) Cardiolipin domains in *Bacillus subtilis* marburg membranes. J Bacteriol 186: 1475–1483 14973018 10.1128/JB.186.5.1475-1483.2004 PMC344405

[embj2020105753-bib-0082] Ke FFS , Vanyai HK , Cowan AD , Delbridge ARD , Whitehead L , Grabow S , Czabotar PE , Voss AK , Strasser A (2018) Embryogenesis and adult life in the absence of intrinsic apoptosis effectors BAX, BAK, and BOK. Cell 173: 1217–1230.e1217 29775594 10.1016/j.cell.2018.04.036

[embj2020105753-bib-0083] Kim J , Gupta R , Blanco LP , Yang S , Shteinfer‐Kuzmine A , Wang K , Zhu J , Yoon HE , Wang X , Kerkhofs M *et al* (2019) VDAC oligomers form mitochondrial pores to release mtDNA fragments and promote lupus‐like disease. Science 366: 1531–1536 31857488 10.1126/science.aav4011 PMC8325171

[embj2020105753-bib-0084] Kuang S , Zheng J , Yang H , Li S , Duan S , Shen Y , Ji C , Gan J , Xu XW , Li J (2017) Structure insight of GSDMD reveals the basis of GSDMD autoinhibition in cell pyroptosis. Proc Natl Acad Sci USA 114: 10642–10647 28928145 10.1073/pnas.1708194114 PMC5635896

[embj2020105753-bib-0085] Kunzelmann K (2016) Ion channels in regulated cell death. Cell Mol Life Sci 73: 2387–2403 27091155 10.1007/s00018-016-2208-z PMC11108559

[embj2020105753-bib-0086] Kushnareva Y , Andreyev AY , Kuwana T , Newmeyer DD (2012) Bax activation initiates the assembly of a multimeric catalyst that facilitates Bax pore formation in mitochondrial outer membranes. PLoS Biol 10: e1001394 23049480 10.1371/journal.pbio.1001394 PMC3457932

[embj2020105753-bib-0087] Kuwana T , Mackey MR , Perkins G , Ellisman MH , Latterich M , Schneiter R , Green DR , Newmeyer DD (2002) Bid, Bax, and lipids cooperate to form supramolecular openings in the outer mitochondrial membrane. Cell 111: 331–342 12419244 10.1016/s0092-8674(02)01036-x

[embj2020105753-bib-0088] Kuwana T , Olson NH , Kiosses WB , Peters B , Newmeyer DD (2016) Pro‐apoptotic Bax molecules densely populate the edges of membrane pores. Sci Rep 6: 27299 27255832 10.1038/srep27299 PMC4891688

[embj2020105753-bib-0089] Landeta O , Landajuela A , Gil D , Taneva S , Di Primo C , Sot B , Valle M , Frolov VA , Basanez G (2011) Reconstitution of proapoptotic BAK function in liposomes reveals a dual role for mitochondrial lipids in the BAK‐driven membrane permeabilization process. J Biol Chem 286: 8213–8230 21196599 10.1074/jbc.M110.165852 PMC3048708

[embj2020105753-bib-0090] Landeta O , Garcia Valero J , Flores‐Romero H , Bustillo‐Zabalbeitia I , Landajuela A , Garcia‐Porras M , Terrones O , Basanez G (2014) Lipid‐dependent bimodal MCL1 membrane activity. ACS Chem Biol 9: 2852–2863 25314294 10.1021/cb500592e

[embj2020105753-bib-0091] Landeta O , Landajuela A , Garcia‐Saez A , Basanez G (2015) Minimalist model systems reveal similarities and differences between membrane interaction modes of MCL1 and BAK. J Biol Chem 290: 17004–17019 25987560 10.1074/jbc.M114.602193 PMC4505444

[embj2020105753-bib-0092] Lauterwasser J , Todt F , Zerbes RM , Nguyen TN , Craigen W , Lazarou M , van der Laan M , Edlich F (2016) The porin VDAC2 is the mitochondrial platform for Bax retrotranslocation. Sci Rep 6: 32994 27620692 10.1038/srep32994 PMC5020405

[embj2020105753-bib-0093] Lee MT , Chen FY , Huang HW (2004) Energetics of pore formation induced by membrane active peptides. Biochemistry 43: 3590–3599 15035629 10.1021/bi036153r

[embj2020105753-bib-0094] Lee CC , Sun Y , Qian S , Huang HW (2011) Transmembrane pores formed by human antimicrobial peptide LL‐37. Biophys J 100: 1688–1696 21463582 10.1016/j.bpj.2011.02.018 PMC3072607

[embj2020105753-bib-0095] Liu X , Zhang Z , Ruan J , Pan Y , Magupalli VG , Wu H , Lieberman J (2016) Inflammasome‐activated gasdermin D causes pyroptosis by forming membrane pores. Nature 535: 153–158 27383986 10.1038/nature18629 PMC5539988

[embj2020105753-bib-0096] Liu S , Liu H , Johnston A , Hanna‐Addams S , Reynoso E , Xiang Y , Wang Z (2017) MLKL forms disulfide bond‐dependent amyloid‐like polymers to induce necroptosis. Proc Natl Acad Sci USA 114: E7450–E7459 28827318 10.1073/pnas.1707531114 PMC5594682

[embj2020105753-bib-0097] Liu Z , Wang C , Rathkey JK , Yang J , Dubyak GR , Abbott DW , Xiao TS (2018) Structures of the gasdermin D C‐terminal domains reveal mechanisms of autoinhibition. Structure 26: 778–784.e3 29576317 10.1016/j.str.2018.03.002 PMC5932255

[embj2020105753-bib-0098] Liu Z , Wang C , Yang J , Zhou B , Yang R , Ramachandran R , Abbott DW , Xiao TS (2019) Crystal structures of the full‐length murine and human gasdermin D reveal mechanisms of autoinhibition, lipid binding, and oligomerization. Immunity 51: 43–49.e4 31097341 10.1016/j.immuni.2019.04.017 PMC6640092

[embj2020105753-bib-0099] Ludtke SJ , He K , Heller WT , Harroun TA , Yang L , Huang HW (1996) Membrane pores induced by magainin. Biochemistry 35: 13723–13728 8901513 10.1021/bi9620621

[embj2020105753-bib-0100] Mandal T , Shin S , Aluvila S , Chen HC , Grieve C , Choe JY , Cheng EH , Hustedt EJ , Oh KJ (2016) Assembly of Bak homodimers into higher order homooligomers in the mitochondrial apoptotic pore. Sci Rep 6: 30763 27488021 10.1038/srep30763 PMC4973285

[embj2020105753-bib-0101] Matsuzaki K , Murase O , Fujii N , Miyajima K (1996) An antimicrobial peptide, magainin 2, induced rapid flip‐flop of phospholipids coupled with pore formation and peptide translocation. Biochemistry 35: 11361–11368 8784191 10.1021/bi960016v

[embj2020105753-bib-0102] McArthur K , Whitehead LW , Heddleston JM , Li L , Padman BS , Oorschot V , Geoghegan ND , Chappaz S , Davidson S , San Chin H *et al* (2018) BAK/BAX macropores facilitate mitochondrial herniation and mtDNA efflux during apoptosis. Science 359: eaao6047 29472455 10.1126/science.aao6047

[embj2020105753-bib-0103] McCormack R , de Armas L , Shiratsuchi M , Podack ER (2013) Killing machines: three pore‐forming proteins of the immune system. Immunol Res 57: 268–278 24293008 10.1007/s12026-013-8469-9 PMC3980504

[embj2020105753-bib-0104] McNamara DE , Dovey CM , Hale AT , Quarato G , Grace CR , Guibao CD , Diep J , Nourse A , Cai CR , Wu H *et al* (2019) Direct activation of human MLKL by a select repertoire of inositol phosphate metabolites. Cell Chem Biol 26: 863–877.e867 31031142 10.1016/j.chembiol.2019.03.010 PMC6588482

[embj2020105753-bib-0105] Mesa‐Galloso H , Valiente PA , Valdes‐Tresanco ME , Epand RF , Lanio ME , Epand RM , Alvarez C , Tieleman DP , Ros U (2019) Membrane remodeling by the lytic fragment of sticholysin II: implications for the toroidal pore model. Biophys J 117: 1563–1576 31587828 10.1016/j.bpj.2019.09.018 PMC6838749

[embj2020105753-bib-0106] Metkar SS , Marchioretto M , Antonini V , Lunelli L , Wang B , Gilbert RJ , Anderluh G , Roth R , Pooga M , Pardo J *et al* (2015) Perforin oligomers form arcs in cellular membranes: a locus for intracellular delivery of granzymes. Cell Death Differ 22: 74–85 25146929 10.1038/cdd.2014.110 PMC4262768

[embj2020105753-bib-0107] Minn AJ , Velez P , Schendel SL , Liang H , Muchmore SW , Fesik SW , Fill M , Thompson CB (1997) Bcl‐x(L) forms an ion channel in synthetic lipid membranes. Nature 385: 353–357 9002522 10.1038/385353a0

[embj2020105753-bib-0108] Moldoveanu T , Grace CR , Llambi F , Nourse A , Fitzgerald P , Gehring K , Kriwacki RW , Green DR (2013) BID‐induced structural changes in BAK promote apoptosis. Nat Struct Mol Biol 20: 589–597 23604079 10.1038/nsmb.2563 PMC3683554

[embj2020105753-bib-0109] Moldoveanu T , Czabotar PE (2019) BAX, BAK, and BOK: a coming of age for the BCL‐2 family effector proteins. Cold Spring Harb Perspect Biol 12: a036319 10.1101/cshperspect.a036319 PMC7111251 31570337

[embj2020105753-bib-0110] Muchmore SW , Sattler M , Liang H , Meadows RP , Harlan JE , Yoon HS , Nettesheim D , Chang BS , Thompson CB , Wong SL *et al* (1996) X‐ray and NMR structure of human Bcl‐xL, an inhibitor of programmed cell death. Nature 381: 335–341 8692274 10.1038/381335a0

[embj2020105753-bib-0111] Mulvihill E , Sborgi L , Mari SA , Pfreundschuh M , Hiller S , Muller DJ (2018) Mechanism of membrane pore formation by human gasdermin‐D. EMBO J 37: e98321 29898893 10.15252/embj.201798321 PMC6043855

[embj2020105753-bib-0112] Murphy JM , Czabotar PE , Hildebrand JM , Lucet IS , Zhang JG , Alvarez‐Diaz S , Lewis R , Lalaoui N , Metcalf D , Webb AI *et al* (2013) The pseudokinase MLKL mediates necroptosis via a molecular switch mechanism. Immunity 39: 443–453 24012422 10.1016/j.immuni.2013.06.018

[embj2020105753-bib-0113] Najafov A , Mookhtiar AK , Luu HS , Ordureau A , Pan H , Amin PP , Li Y , Lu Q , Yuan J (2019) TAM kinases promote necroptosis by regulating oligomerization of MLKL. Mol Cell 75: 457–468.e4 31230815 10.1016/j.molcel.2019.05.022

[embj2020105753-bib-0114] Nechushtan A , Smith CL , Hsu YT , Youle RJ (1999) Conformation of the Bax C‐terminus regulates subcellular location and cell death. EMBO J 18: 2330–2341 10228148 10.1093/emboj/18.9.2330 PMC1171316

[embj2020105753-bib-0115] Nechushtan A , Smith CL , Lamensdorf I , Yoon SH , Youle RJ (2001) Bax and Bak coalesce into novel mitochondria‐associated clusters during apoptosis. J Cell Biol 153: 1265–1276 11402069 10.1083/jcb.153.6.1265 PMC2192024

[embj2020105753-bib-0116] Ortiz A , Killian JA , Verkleij AJ , Wilschut J (1999) Membrane fusion and the lamellar‐to‐inverted‐hexagonal phase transition in cardiolipin vesicle systems induced by divalent cations. Biophys J 77: 2003–2014 10512820 10.1016/S0006-3495(99)77041-4 PMC1300481

[embj2020105753-bib-0117] Ousingsawat J , Cabrita I , Wanitchakool P , Sirianant L , Krautwald S , Linkermann A , Schreiber R , Kunzelmann K (2017) Ca(2+) signals, cell membrane disintegration, and activation of TMEM16F during necroptosis. Cell Mol Life Sci 74: 173–181 27535660 10.1007/s00018-016-2338-3 PMC11107605

[embj2020105753-bib-0118] Parisi LR , Morrow LM , Visser MB , Atilla‐Gokcumen GE (2018) Turning the spotlight on lipids in non‐apoptotic cell death. ACS Chem Biol 13: 506–515 29376324 10.1021/acschembio.7b01082

[embj2020105753-bib-0119] Petrie EJ , Hildebrand JM , Murphy JM (2017) Insane in the membrane: a structural perspective of MLKL function in necroptosis. Immunol Cell Biol 95: 152–159 27999433 10.1038/icb.2016.125

[embj2020105753-bib-0120] Petrie EJ , Sandow JJ , Jacobsen AV , Smith BJ , Griffin MDW , Lucet IS , Dai W , Young SN , Tanzer MC , Wardak A *et al* (2018) Conformational switching of the pseudokinase domain promotes human MLKL tetramerization and cell death by necroptosis. Nat Commun 9: 2422 29930286 10.1038/s41467-018-04714-7 PMC6013482

[embj2020105753-bib-0121] Petrie EJ , Birkinshaw RW , Koide A , Denbaum E , Hildebrand JM , Garnish SE , Davies KA , Sandow JJ , Samson AL , Gavin X *et al* (2020) Identification of MLKL membrane translocation as a checkpoint in necroptotic cell death using Monobodies. Proc Natl Acad Sci USA 117: 8468–8475 32234780 10.1073/pnas.1919960117 PMC7165463

[embj2020105753-bib-0122] Petros AM , Olejniczak ET , Fesik SW (2004) Structural biology of the Bcl‐2 family of proteins. Biochem Biophys Acta 1644: 83–94 14996493 10.1016/j.bbamcr.2003.08.012

[embj2020105753-bib-0123] Podobnik M , Savory P , Rojko N , Kisovec M , Wood N , Hambley R , Pugh J , Wallace EJ , McNeill L , Bruce M *et al* (2016) Crystal structure of an invertebrate cytolysin pore reveals unique properties and mechanism of assembly. Nat Commun 7: 11598 27176125 10.1038/ncomms11598 PMC4865846

[embj2020105753-bib-0124] Prinz D , Klein K , List J , Knab VM , Menzl I , Leidenfrost N , Heller G , Polic B , Putz EM , Witalisz‐Siepracka A *et al* (2020) Loss of NKG2D in murine NK cells leads to increased perforin production upon long‐term stimulation with IL‐2. Eur J Immunol 50: 880–890 32052406 10.1002/eji.201948222 PMC7318224

[embj2020105753-bib-0125] Puech PH , Borghi N , Karatekin E , Brochard‐Wyart F (2003) Line thermodynamics: adsorption at a membrane edge. Phys Rev Lett 90: 128304 12688911 10.1103/PhysRevLett.90.128304

[embj2020105753-bib-0126] Qian S , Wang W , Yang L , Huang HW (2008) Structure of transmembrane pore induced by Bax‐derived peptide: evidence for lipidic pores. Proc Natl Acad Sci USA 105: 17379–17383 18987313 10.1073/pnas.0807764105 PMC2582298

[embj2020105753-bib-0127] Quarato G , Guy CS , Grace CR , Llambi F , Nourse A , Rodriguez DA , Wakefield R , Frase S , Moldoveanu T , Green DR (2016) Sequential engagement of distinct MLKL phosphatidylinositol‐binding sites executes necroptosis. Mol Cell 61: 589–601 26853145 10.1016/j.molcel.2016.01.011 PMC4769881

[embj2020105753-bib-0128] Rehm M , Dussmann H , Prehn JH (2003) Real‐time single cell analysis of Smac/DIABLO release during apoptosis. J Cell Biol 162: 1031–1043 12975347 10.1083/jcb.200303123 PMC2172837

[embj2020105753-bib-0129] Reynwar BJ , Illya G , Harmandaris VA , Muller MM , Kremer K , Deserno M (2007) Aggregation and vesiculation of membrane proteins by curvature‐mediated interactions. Nature 447: 461–464 17522680 10.1038/nature05840

[embj2020105753-bib-0130] Riley JS , Quarato G , Cloix C , Lopez J , O'Prey J , Pearson M , Chapman J , Sesaki H , Carlin LM , Passos JF *et al* (2018) Mitochondrial inner membrane permeabilisation enables mtDNA release during apoptosis. EMBO J 37: e99238 30049712 10.15252/embj.201899238 PMC6120664

[embj2020105753-bib-0131] Rojko N , Anderluh G (2015) How lipid membranes affect pore forming toxin activity. Acc Chem Res 48: 3073–3079 26641659 10.1021/acs.accounts.5b00403

[embj2020105753-bib-0132] Ros U , Garcia‐Saez AJ (2015) More than a pore: the interplay of pore‐forming proteins and lipid membranes. J Membr Biol 248: 545–561 26087906 10.1007/s00232-015-9820-y

[embj2020105753-bib-0133] Ros U , Pena‐Blanco A , Hanggi K , Kunzendorf U , Krautwald S , Wong WW , Garcia‐Saez AJ (2017) Necroptosis execution is mediated by plasma membrane nanopores independent of calcium. Cell Rep 19: 175–187 28380356 10.1016/j.celrep.2017.03.024 PMC5465952

[embj2020105753-bib-0134] Ros U , Pedrera L , Garcia‐Saez AJ (2020) Partners in crime: the interplay of proteins and membranes in regulated necrosis. Int J Mol Sci 21: 2412 10.3390/ijms21072412 PMC7177786 32244433

[embj2020105753-bib-0135] Ruan J , Xia S , Liu X , Lieberman J , Wu H (2018) Cryo‐EM structure of the gasdermin A3 membrane pore. Nature 557: 62–67 29695864 10.1038/s41586-018-0058-6 PMC6007975

[embj2020105753-bib-0136] Ruhl S , Shkarina K , Demarco B , Heilig R , Santos JC , Broz P (2018) ESCRT‐dependent membrane repair negatively regulates pyroptosis downstream of GSDMD activation. Science 362: 956–960 30467171 10.1126/science.aar7607

[embj2020105753-bib-0137] Salvador‐Gallego R , Mund M , Cosentino K , Schneider J , Unsay J , Schraermeyer U , Engelhardt J , Ries J , Garcia‐Saez AJ (2016) Bax assembly into rings and arcs in apoptotic mitochondria is linked to membrane pores. EMBO J 35: 389–401 26783362 10.15252/embj.201593384 PMC4755116

[embj2020105753-bib-0138] Samson AL , Zhang Y , Geoghegan ND , Gavin XJ , Davies KA , Mlodzianoski MJ , Whitehead LW , Frank D , Garnish SE , Fitzgibbon C *et al* (2020) MLKL trafficking and accumulation at the plasma membrane control the kinetics and threshold for necroptosis. Nat Commun 11: 3151 32561730 10.1038/s41467-020-16887-1 PMC7305196

[embj2020105753-bib-0139] Sattler M , Liang H , Nettesheim D , Meadows RP , Harlan JE , Eberstadt M , Yoon HS , Shuker SB , Chang BS , Minn AJ *et al* (1997) Structure of Bcl‐xL‐Bak peptide complex: recognition between regulators of apoptosis. Science 275: 983–986 9020082 10.1126/science.275.5302.983

[embj2020105753-bib-0140] Sborgi L , Ruhl S , Mulvihill E , Pipercevic J , Heilig R , Stahlberg H , Farady CJ , Muller DJ , Broz P , Hiller S (2016) GSDMD membrane pore formation constitutes the mechanism of pyroptotic cell death. EMBO J 35: 1766–1778 27418190 10.15252/embj.201694696 PMC5010048

[embj2020105753-bib-0141] Schellenberg B , Wang P , Keeble JA , Rodriguez‐Enriquez R , Walker S , Owens TW , Foster F , Tanianis‐Hughes J , Brennan K , Streuli CH *et al* (2013) Bax exists in a dynamic equilibrium between the cytosol and mitochondria to control apoptotic priming. Mol Cell 49: 959–971 23375500 10.1016/j.molcel.2012.12.022 PMC3594749

[embj2020105753-bib-0142] Schlame M , Ren M (2009) The role of cardiolipin in the structural organization of mitochondrial membranes. Biochem Biophys Acta 1788: 2080–2083 19413994 10.1016/j.bbamem.2009.04.019 PMC2757492

[embj2020105753-bib-0143] Schlesinger PH , Gross A , Yin XM , Yamamoto K , Saito M , Waksman G , Korsmeyer SJ (1997) Comparison of the ion channel characteristics of proapoptotic BAX and antiapoptotic BCL‐2. Proc Natl Acad Sci USA 94: 11357–11362 9326614 10.1073/pnas.94.21.11357 PMC23466

[embj2020105753-bib-0144] Schwarz G , Robert CH (1992) Kinetics of pore‐mediated release of marker molecules from liposomes or cells. Biophys Chem 42: 291–296 1581523 10.1016/0301-4622(92)80021-v

[embj2020105753-bib-0145] Sengupta D , Leontiadou H , Mark AE , Marrink SJ (2008) Toroidal pores formed by antimicrobial peptides show significant disorder. Biochim Biophys Acta 1778: 2308–2317 18602889 10.1016/j.bbamem.2008.06.007

[embj2020105753-bib-0146] Shlomovitz R , Gov NS (2009) Membrane‐mediated interactions drive the condensation and coalescence of FtsZ rings. Phys Biol 6: 046017 10.1088/1478-3975/6/4/046017 19934492

[embj2020105753-bib-0147] Singh R , Letai A , Sarosiek K (2019) Regulation of apoptosis in health and disease: the balancing act of BCL‐2 family proteins. Nat Rev Mol Cell Biol 20: 175–193 30655609 10.1038/s41580-018-0089-8 PMC7325303

[embj2020105753-bib-0148] Sonnen AF , Plitzko JM , Gilbert RJ (2014) Incomplete pneumolysin oligomers form membrane pores. Open Biol 4: 140044 24759615 10.1098/rsob.140044 PMC4043118

[embj2020105753-bib-0149] Sorice M , Manganelli V , Matarrese P , Tinari A , Misasi R , Malorni W , Garofalo T (2009) Cardiolipin‐enriched raft‐like microdomains are essential activating platforms for apoptotic signals on mitochondria. FEBS Lett 583: 2447–2450 19616549 10.1016/j.febslet.2009.07.018

[embj2020105753-bib-0150] Steringer JP , Bleicken S , Andreas H , Zacherl S , Laussmann M , Temmerman K , Contreras FX , Bharat TA , Lechner J , Muller HM *et al* (2012) Phosphatidylinositol 4,5‐bisphosphate (PI(4,5)P2)‐dependent oligomerization of fibroblast growth factor 2 (FGF2) triggers the formation of a lipidic membrane pore implicated in unconventional secretion. J Biol Chem 287: 27659–27669 22730382 10.1074/jbc.M112.381939 PMC3431657

[embj2020105753-bib-0151] Su L , Quade B , Wang H , Sun L , Wang X , Rizo J (2014) A plug release mechanism for membrane permeation by MLKL. Structure 22: 1489–1500 25220470 10.1016/j.str.2014.07.014 PMC4192069

[embj2020105753-bib-0152] Subburaj Y , Cosentino K , Axmann M , Pedrueza‐Villalmanzo E , Hermann E , Bleicken S , Spatz J , Garcia‐Saez AJ (2015) Bax monomers form dimer units in the membrane that further self‐assemble into multiple oligomeric species. Nat Commun 6: 8042 26271728 10.1038/ncomms9042 PMC4557355

[embj2020105753-bib-0153] Suzuki M , Youle RJ , Tjandra N (2000) Structure of Bax: coregulation of dimer formation and intracellular localization. Cell 103: 645–654 11106734 10.1016/s0092-8674(00)00167-7

[embj2020105753-bib-0154] Tanzer MC , Tripaydonis A , Webb AI , Young SN , Varghese LN , Hall C , Alexander WS , Hildebrand JM , Silke J , Murphy JM (2015) Necroptosis signalling is tuned by phosphorylation of MLKL residues outside the pseudokinase domain activation loop. Biochem J 471: 255–265 26283547 10.1042/BJ20150678

[embj2020105753-bib-0155] Tanzer MC , Matti I , Hildebrand JM , Young SN , Wardak A , Tripaydonis A , Petrie EJ , Mildenhall AL , Vaux DL , Vince JE *et al* (2016) Evolutionary divergence of the necroptosis effector MLKL. Cell Death Differ 23: 1185–1197 26868910 10.1038/cdd.2015.169 PMC4946887

[embj2020105753-bib-0156] Tayeb‐Fligelman E , Tabachnikov O , Moshe A , Goldshmidt‐Tran O , Sawaya MR , Coquelle N , Colletier JP , Landau M (2017) The cytotoxic *Staphylococcus aureus*PSMalpha3 reveals a cross‐alpha amyloid‐like fibril. Science 355: 831–833 28232575 10.1126/science.aaf4901 PMC6372758

[embj2020105753-bib-0157] Terrones O , Antonsson B , Yamaguchi H , Wang HG , Liu J , Lee RM , Herrmann A , Basanez G (2004) Lipidic pore formation by the concerted action of proapoptotic BAX and tBID. J Biol Chem 279: 30081–30091 15138279 10.1074/jbc.M313420200

[embj2020105753-bib-0158] Tieleman DP , Leontiadou H , Mark AE , Marrink SJ (2003) Simulation of pore formation in lipid bilayers by mechanical stress and electric fields. J Am Chem Soc 125: 6382–6383 12785774 10.1021/ja029504i

[embj2020105753-bib-0159] Tieleman DP , Marrink SJ (2006) Lipids out of equilibrium: energetics of desorption and pore mediated flip‐flop. J Am Chem Soc 128: 12462–12467 16984196 10.1021/ja0624321

[embj2020105753-bib-0160] Todt F , Cakir Z , Reichenbach F , Emschermann F , Lauterwasser J , Kaiser A , Ichim G , Tait SW , Frank S , Langer HF *et al* (2015) Differential retrotranslocation of mitochondrial Bax and Bak. EMBO J 34: 67–80 25378477 10.15252/embj.201488806 PMC4291481

[embj2020105753-bib-0161] Ugarte‐Uribe B , Garcia‐Saez AJ (2017) Apoptotic foci at mitochondria: in and around Bax pores. Philos Trans R Soc B Biol Sci 372: 20160217 10.1098/rstb.2016.0217 PMC5483519 28630156

[embj2020105753-bib-0162] Ugarte‐Uribe B , Prevost C , Das KK , Bassereau P , Garcia‐Saez AJ (2018) Drp1 polymerization stabilizes curved tubular membranes similar to those of constricted mitochondria. J Cell Sci 132: jcs208603 29361534 10.1242/jcs.208603

[embj2020105753-bib-0163] Unsay JD , Cosentino K , Subburaj Y , Garcia‐Saez AJ (2013) Cardiolipin effects on membrane structure and dynamics. Langmuir 29: 15878–15887 23962277 10.1021/la402669z

[embj2020105753-bib-0164] Unsay JD , Cosentino K , Sporbeck K , Garcia‐Saez AJ (2017) Pro‐apoptotic cBid and Bax exhibit distinct membrane remodeling activities: An AFM study. Biochim Biophys Acta 1859: 17–27 10.1016/j.bbamem.2016.10.007 27755971

[embj2020105753-bib-0165] Valcarcel CA , Dalla Serra M , Potrich C , Bernhart I , Tejuca M , Martinez D , Pazos F , Lanio ME , Menestrina G (2001) Effects of lipid composition on membrane permeabilization by sticholysin I and II, two cytolysins of the sea anemone Stichodactyla helianthus. Biophys J 80: 2761–2774 11371451 10.1016/S0006-3495(01)76244-3 PMC1301462

[embj2020105753-bib-0166] Vance JE (1990) Phospholipid synthesis in a membrane fraction associated with mitochondria. J Biol Chem 265: 7248–7256 2332429

[embj2020105753-bib-0167] Voskoboinik I , Whisstock JC , Trapani JA (2015) Perforin and granzymes: function, dysfunction and human pathology. Nat Rev Immunol 15: 388–400 25998963 10.1038/nri3839

[embj2020105753-bib-0168] Wallach D , Kang TB , Dillon CP , Green DR (2016) Programmed necrosis in inflammation: toward identification of the effector molecules. Science 352: aaf2154 27034377 10.1126/science.aaf2154

[embj2020105753-bib-0169] Westphal D , Dewson G , Menard M , Frederick P , Iyer S , Bartolo R , Gibson L , Czabotar PE , Smith BJ , Adams JM *et al* (2014) Apoptotic pore formation is associated with in‐plane insertion of Bak or Bax central helices into the mitochondrial outer membrane. Proc Natl Acad Sci USA 111: E4076–E4085 25228770 10.1073/pnas.1415142111 PMC4191798

[embj2020105753-bib-0170] Woo SY , Lee H (2017) Effect of lipid shape on toroidal pore formation and peptide orientation in lipid bilayers. Phys Chem Chem Phys 19: 21340–21349 28762427 10.1039/c7cp02708g

[embj2020105753-bib-0171] Xia B , Fang S , Chen X , Hu H , Chen P , Wang H , Gao Z (2016) MLKL forms cation channels. Cell Res 26: 517–528 27033670 10.1038/cr.2016.26 PMC4856759

[embj2020105753-bib-0172] Xu XP , Zhai D , Kim E , Swift M , Reed JC , Volkmann N , Hanein D (2013) Three‐dimensional structure of Bax‐mediated pores in membrane bilayers. Cell Death Dis 4: e683 23788040 10.1038/cddis.2013.210 PMC3702287

[embj2020105753-bib-0173] Yamaguchi R , Lartigue L , Perkins G , Scott RT , Dixit A , Kushnareva Y , Kuwana T , Ellisman MH , Newmeyer DD (2008) Opa1‐mediated cristae opening is Bax/Bak and BH3 dependent, required for apoptosis, and independent of Bak oligomerization. Mol Cell 31: 557–569 18691924 10.1016/j.molcel.2008.07.010 PMC2636708

[embj2020105753-bib-0174] Yang L , Harroun TA , Weiss TM , Ding L , Huang HW (2001) Barrel‐stave model or toroidal model? A case study on melittin pores. Biophys J 81: 1475–1485 11509361 10.1016/S0006-3495(01)75802-X PMC1301626

[embj2020105753-bib-0175] Yoon S , Kovalenko A , Bogdanov K , Wallach D (2017) MLKL, the protein that mediates necroptosis, also regulates endosomal trafficking and extracellular vesicle generation. Immunity 47: 51–65.e7 28666573 10.1016/j.immuni.2017.06.001

[embj2020105753-bib-0176] Youle RJ , Strasser A (2008) The BCL‐2 protein family: opposing activities that mediate cell death. Nat Rev Mol Cell Biol 9: 47–59 18097445 10.1038/nrm2308

[embj2020105753-bib-0177] Zanoni I , Tan Y , Di Gioia M , Broggi A , Ruan J , Shi J , Donado CA , Shao F , Wu H , Springstead JR *et al* (2016) An endogenous caspase‐11 ligand elicits interleukin‐1 release from living dendritic cells. Science 352: 1232–1236 27103670 10.1126/science.aaf3036 PMC5111085

[embj2020105753-bib-0178] Zeitler M , Steringer JP , Muller HM , Mayer MP , Nickel W (2015) HIV‐Tat protein forms phosphoinositide‐dependent membrane pores implicated in unconventional protein secretion. J Biol Chem 290: 21976–21984 26183781 10.1074/jbc.M115.667097 PMC4571951

[embj2020105753-bib-0179] Zhang Z , Zhu W , Lapolla SM , Miao Y , Shao Y , Falcone M , Boreham D , McFarlane N , Ding J , Johnson AE *et al* (2010) Bax forms an oligomer via separate, yet interdependent, surfaces. J Biol Chem 285: 17614–17627 20382739 10.1074/jbc.M110.113456 PMC2878526

[embj2020105753-bib-0180] Zhou LL , Zhou LY , Luo KQ , Chang DC (2005) Smac/DIABLO and cytochrome *c* are released from mitochondria through a similar mechanism during UV‐induced apoptosis. Apoptosis 10: 289–299 15843890 10.1007/s10495-005-0803-9

